# Dynamic remodeling of the tumor immune microenvironment: immunometabolic regulatory networks of astragalus polysaccharides and astragaloside IV

**DOI:** 10.3389/fphar.2026.1833078

**Published:** 2026-08-26

**Authors:** Ziming Xu, Lijie Wang, Xin Zhang, Yue Qi

**Affiliations:** 1 First Clinical Medical College, Shandong University of Traditional Chinese Medicine, Jinan, China; 2 Shandong Key Laboratory of Digital Traditional Chinese Medicine, Shandong University of Traditional Chinese Medicine, Jinan, China; 3 Institute of Pharmacy, Shandong University of Traditional Chinese Medicine, Jinan, China

**Keywords:** astragaloside IV, astragalus polysaccharides, immunometabolism, metabolic reprogramming, precision immunotherapy, tumor immune microenvironment

## Abstract

The highly heterogeneous tumor immune microenvironment (TIME) remains a major obstacle to effective cancer immunotherapy. This review systematically elucidates the immunometabolic regulatory networks of the primary active constituents of Astragalus membranaceus—Astragalus polysaccharides (APS) and Astragaloside IV (AS-IV)—within the TIME. Synthesizing preclinical evidence up to January 2026, we focus on immune cell modulation, metabolic interventions, and advanced translational strategies. Evidence indicates that APS and AS-IV modulate the TIME through two primary mechanisms: regulating cellular immunity (APS facilitates dendritic cell maturation and attenuates T-cell exhaustion, while AS-IV promotes an immunostimulatory M1 macrophage polarization) and modulating tumor bioenergetics (inhibiting aerobic glycolysis and lipid metabolism to alleviate resource competition). Furthermore, experimental models demonstrate that co-delivering these agents via advanced nanoplatforms or in combination with immune checkpoint inhibitors significantly enhances anti-tumor responses. Ultimately, APS and AS-IV exhibit promising potential to shift the TIME from an immunosuppressive state toward an immunostimulatory microenvironment, highlighting their potential transition from empirical adjuvants to rationally designed precision immunotherapies.

## Graphical Abstract

**Figure:**
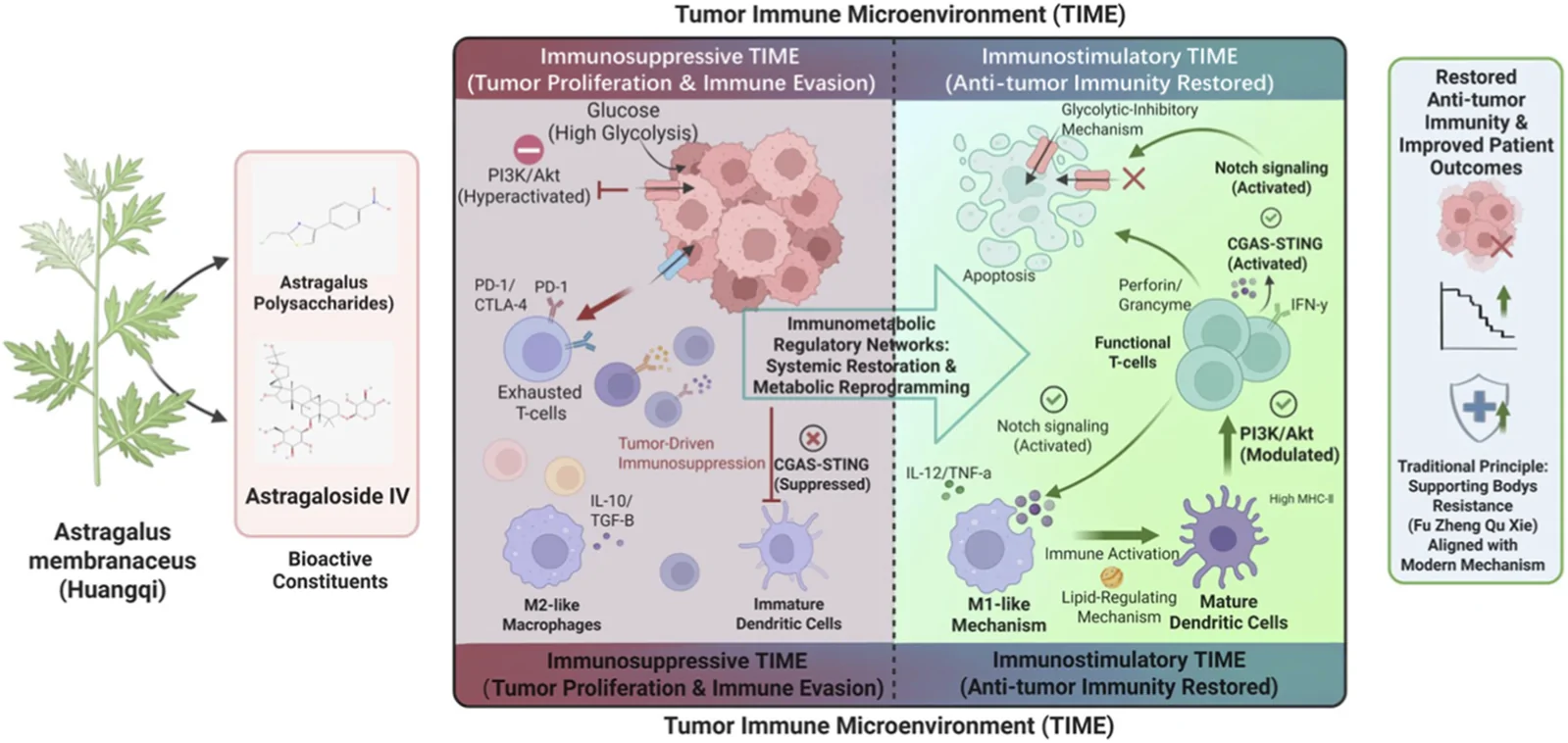

## Highlights

- Immunometabolic modulation: APS and AS-IV regulate the tumor immune microenvironment by integrating immune cell modulation with metabolic pathway interventions.
- Cellular immune regulation: these agents attenuate T-cell exhaustion and promote anti-tumorigenic macrophage polarization via Notch, cGAS-STING, and PI3K/Akt signaling pathways.
- Modulation of tumor bioenergetics: both botanical constituents modulate the Warburg effect and tumor lipid metabolism, potentially alleviating microenvironmental resource competition and supporting the metabolic fitness of immune effectors.
- High-resolution spatiotemporal profiling: future investigations should incorporate advanced single-cell and spatial transcriptomics to characterize the dynamic clonal evolution and spatial architecture of the TIME.
- Strategies for clinical translation: a systematic translational framework is proposed, emphasizing biomarker-guided patient stratification, advanced nano-delivery systems, and high-fidelity patient-derived organoid (PDO) models.

## 1 Introduction

The tumor immune microenvironment (TIME) serves as a critical regulatory niche within the broader structural and cellular framework of the tumor microenvironment (TME). The overarching TME encompasses cellular constituents (tumor, immune, and stromal cells) alongside non-cellular components, such as the extracellular matrix, vasculature, and diverse metabolic provisions (Riera-Domingo et al., 2020; Visser and Joyce, 2023). Within this complex ecosystem, the TIME specifically denotes the immunoregulatory network established by infiltrating immune cells—such as Cluster of Differentiation 8^+^ T cells (CD8^+^ T cells), macrophages, and regulatory T cells (Tregs)—and their dynamic communication via cytokines and chemokines (Bader et al., 2020; Riera-Domingo et al., 2020; Fu et al., 2021). The continuous remodeling of this microenvironmental state is directly linked to tumor formation, dissemination, and patient clinical outcomes (Fu et al., 2019).

Therapeutic strategies targeting the TIME aim to enhance or restore the host’s intrinsic immune responses against the malignancy. This approach offers antigen specificity, a potentially more favorable toxicity profile than conventional chemotherapy, and durable protection through the generation of memory T cells (Lv et al., 2022). Consequently, the development of TIME-focused interventions is accelerating. For example, in preclinical murine breast cancer models, acetate supplementation reinforces T-cell effector function, whereas inhibiting acetyl-CoA synthetase 2 (ACSS2) disrupts the tumor’s metabolic networks, thereby sensitizing tumors to chemotherapy (Miller et al., 2023). While these specific metabolic manipulations show clear preclinical promise, executing them systemically requires careful calibration to avoid disrupting healthy physiological metabolism in humans. Beyond these therapeutic challenges, the host-tumor interaction is fundamentally a bidirectional, dynamic biological process. Malignancies actively modulate their surroundings to reprogram the immune stroma and promote immune evasion. For instance, in triple-negative breast cancer models, cancer-associated fibroblasts (CAFs) can convert recruited monocytes into an immunosuppressive Stabilin 1^+^(STAB1^+^) Triggering Receptor Expressed on Myeloid Cells 2^+^ (TREM2^+^) lipid-associated macrophage (LAM) subset (Jenkins et al., 2022; Timperi et al., 2022). This phenotypic transition suppresses T-cell activation and increases chemotherapy resistance, underscoring the necessity of rationally targeting these TIME dynamics.

These collective immunosuppressive properties essentially function as robust barriers that substantially hinder effective clinical therapy. For instance, alongside the suppressive cellular niches fostered by CAFs, the tumor’s reliance on aerobic glycolysis (the Warburg effect) generates a lactate-rich metabolic milieu that further impairs immune surveillance (Jiang et al., 2022; Ma et al., 2023; Ye et al., 2024). Dissecting the temporal evolution and spatial organization of the TIME during tumor progression is therefore a critical step toward precision immuno-oncology. Still, because current analytical frameworks frequently rely on static biological snapshots, adopting more dynamic assessment models is essential for systematically screening TIME-directed pharmacological agents.

Within traditional herbal medicine, Astragalus membranaceus (Huangqi), the dried root of A. membranaceus var. mongholicus and A. membranaceus, has long served as a therapeutic agent. Modern pharmacological studies have identified diverse biological activities associated with Astragalus, including immunomodulatory (Li et al., 2022), potential anticancer (Sheng et al., 2024), anti-inflammatory (Chen et al., 2023), and antioxidant effects (Shahzad et al., 2016) in various preclinical models. Clinically, it is frequently utilized as an adjuvant in cancer care (Tang and Tian, 2024) and in managing chronic cardiovascular and metabolic diseases (Wu C. et al., 2024). Its principal bioactive constituents comprise Astragalus polysaccharides (APS), Astragaloside IV (AS-IV), and flavonoids (Dan et al., 2024), with APS and AS-IV emerging as key components contributing to its potential antitumor efficacy (Xu et al., 2023). While empirical clinical applications of these compounds are widespread, translating these empirical observations into precision oncology requires a rigorous deconvolution of their precise molecular mechanisms and an objective assessment of their limitations in complex biological systems.

In this regard, APS and AS-IV appear to modulate the TIME through distinct yet complementary mechanisms. APS primarily functions as a regulatory agent for T- and B-cell immunity. It dose-dependently expands the populations of Cluster of Differentiation 3^+^ T cells (CD3^+^ T cells), Cluster of Differentiation 4^+^ T cells (CD4^+^ T cells), CD8^+^ T cells, and Cluster of Differentiation 19^+^ B cells (CD19^+^ B cells) in the thymus, peripheral blood, and spleen of Sarcoma 180 (S180) tumor-bearing mice (Yu et al., 2021). Similarly, in preclinical models of inflammation-associated colorectal cancer (CRC), APS has been shown to mitigate tumor progression by activating CD8^+^ T cells and attenuating their suppression within the TME (Li Q. et al., 2024). Concurrently, AS-IV serves as a modulator of macrophage polarization. Experimental evidence indicates that AS-IV can inhibit the transition of macrophages toward a pro-tumor M2 phenotype while promoting a shift toward a pro-inflammatory M1 state across various *in vitro* and *in vivo* settings (Wang X. et al., 2021; Wu T. et al., 2024; Zhou et al., 2024). Ultimately, a systematic deconvolution of these synergistic, multi-pathway mechanisms is essential to evaluate the potential of APS and AS-IV as candidates for rationally designed immunotherapeutic agents.

## 2 Search strategy

To systematically review the immunometabolic regulatory effects of APS and AS-IV, we comprehensively analyzed the preclinical literature from database inception up to January 2026. A systematic literature search was conducted utilizing the PubMed and Web of Science (WoS) databases. The search strategy employed a combination of keywords and abbreviations using the following Boolean logic: (“Astragalus polysaccharides” OR “APS” OR “Astragaloside IV” OR “AS-IV”) AND (“tumor microenvironment” OR “TME” OR “tumor immune microenvironment” OR “TIME”). The retrieved literature primarily focused on studies investigating immune cell modulation, metabolic interventions, and the application of advanced translational strategies within the TIME.

## 3 Targeted modulation of TIME core components by APS and AS-IV

### 3.1 Immune cell network

The TIME functions as a highly regulated cellular network integrated within the broader TME. The core cellular components in this microenvironment primarily consist of adaptive immune cells, myeloid lineages, and hybrid innate-like cells, such as innate lymphoid cells (ILCs). Specifically, the adaptive compartment encompasses CD8^+^ T cells, CD4^+^ T cells, Tregs, and B cells. The myeloid populations include macrophages, neutrophils, monocytes, dendritic cells (DCs), mast cells, eosinophils, myeloid-derived suppressor cells (MDSCs), and platelets. Meanwhile, cells exhibiting both innate and adaptive features, such as ILCs, comprise natural killer cells (NK cells), invariant natural killer T cells (iNKT cells), and γδ T cells, among others (Visser and Joyce, 2023).

Based on their functional roles in tumor progression, these diverse cell types can be broadly classified into two distinct categories. The tumor-suppressive populations mainly involve effector T cells (cytotoxic CD8^+^ T cells and effector CD4^+^ T cells), NK cells, DCs, and M1-type macrophages. Conversely, the pro-tumor subsets include Tregs, MDSCs, M2-type macrophages, N2-type neutrophils, type 2 natural killer T (NKT2) cells, and type 2 innate lymphoid cells (ILC2s) (Lv et al., 2022). Furthermore, these functional classifications are fluid rather than rigid. The dynamic states of these cells are continuously reshaped by local microenvironmental factors, including antigen load, the cytokine milieu, stromal cross-talk, and specific metabolic constraints within the TME.

#### 3.1.1 Enhancement of anti-tumor immune cell functions

##### 3.1.1.1 Mitigation of T-Cell exhaustion and metabolic reinvigoration

**FIGURE 1:**
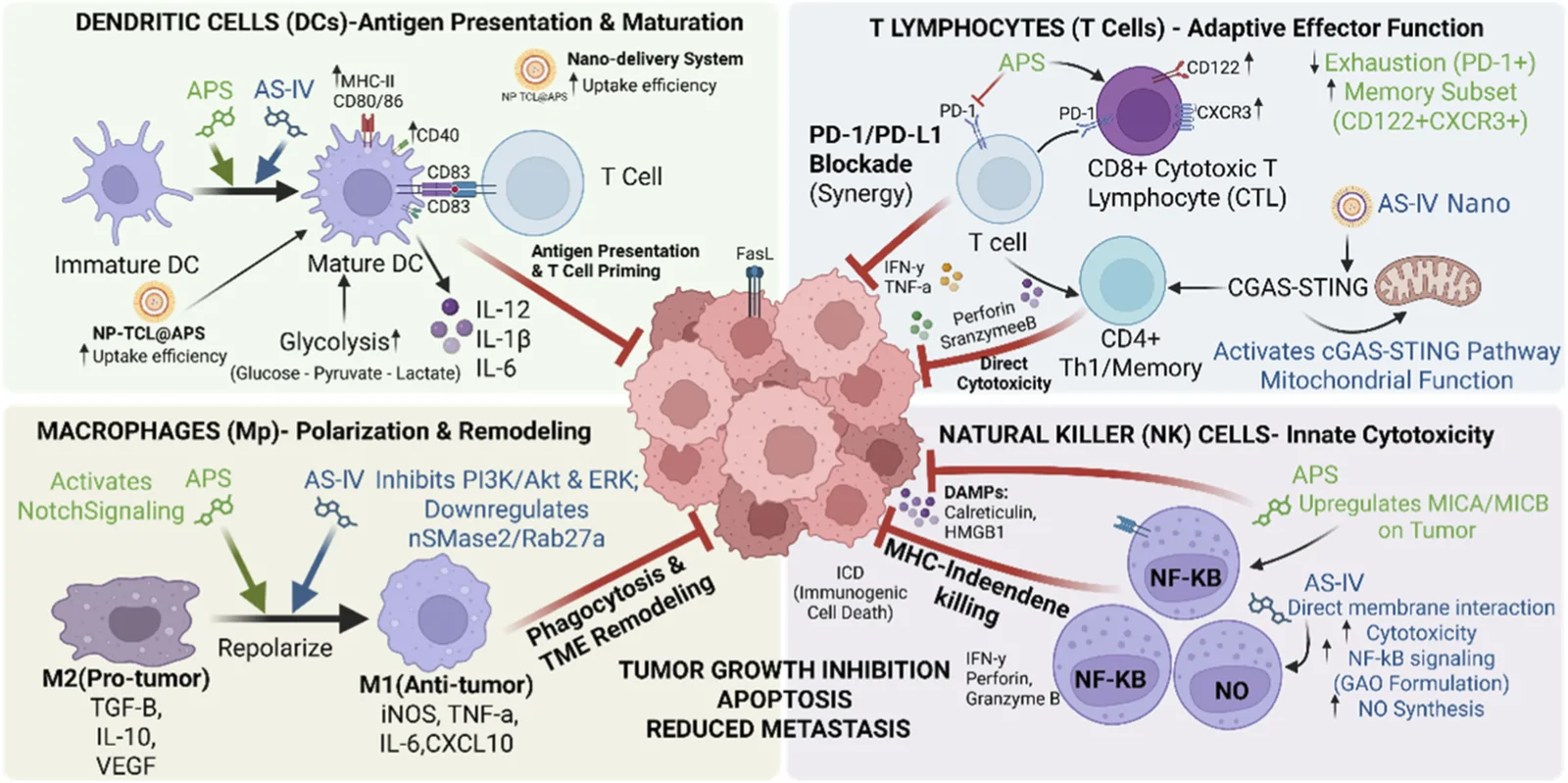
The immunomodulatory network of APS and AS-IV in remodeling the TIME. This schematic maps the distinct and complementary pathways modulated by APS (green pathways) and AS-IV (blue pathways) across diverse immune populations within the TME. During antigen presentation, APS is reported to promote DC maturation through glycolytic metabolic reprogramming, an effect further augmented when incorporated into engineered nanoparticle systems. Meanwhile, AS-IV upregulates essential costimulatory molecules (CD80/86, CD40, CD83, HLA-DR) and IL-12 secretion, collectively supporting the robust priming of naive T cells in preclinical models. Within the T-lymphocyte compartment, APS mitigates exhaustion by reducing the PD-1^+^ subset and expanding the CD122^+^CXCR3^+^ memory T-cell reservoir, thereby attenuating the PD-1/PD-L1 inhibitory axis. Concurrently, when utilized within multi-agent nano-formulations, AS-IV contributes to the restoration of mitochondrial metabolism in CTLs, while co-delivered components trigger the cGAS-STING pathway. This combinatorial approach synergizes with immune checkpoint blockade to enhance cytotoxicity via the release of IFN-γ, TNF-α, perforin, and granzyme B. Regarding innate immunity, APS potentiates NK cell cytotoxicity by upregulating MICA/MICB ligands on tumor surfaces and modulating NO synthesis. Parallelly, AS-IV activates NF-κB signaling in NK cells to increase the production of effector molecules. Furthermore, both agents modulate the myeloid compartment toward an anti-tumorigenic M1 phenotype: APS engages the Notch signaling cascade to drive the secretion of pro-inflammatory mediators (iNOS, TNF-α, IL-6, CXCL10), whereas AS-IV suppresses M2 polarization by inhibiting the PI3K/Akt and ERK signaling pathways and downregulating nSMase2/Rab27a-mediated exosome secretion. Ultimately, this coordinated multi-cellular modulation enhances tumor antigen recognition, induces ICD, and drives tumor cell apoptosis, thereby impeding tumor growth and metastasis in experimental settings.

Tumor-infiltrating lymphocytes (TILs) serve as the primary mediators of anti-tumor immunity (Li et al., 2023), relying on CD8^+^ T cells as direct cytotoxic effectors (Ahrends and Borst, 2018) and CD4^+^ T cells to both orchestrate innate immunity and mediate cytotoxicity against human leukocyte antigen class II (HLA class II)-expressing tumors via the secretion of interferon-gamma (IFN-γ) and tumor necrosis factor (TNF) (Braumüller et al., 2013). This tumor clearance is primarily achieved through apoptosis driven by pro-inflammatory cytokines, Fas Ligand (Fas/FasL) interactions, and perforin-mediated cytotoxic granules (Schmidt and Varga, 2018). However, the TME actively suppresses these mechanisms by upregulating inhibitory checkpoints to induce T-cell functional exhaustion (Li et al., 2023), making the blockade of negative regulatory nodes like programmed death-ligand 1 (PD-L1) a fundamental strategy to reinvigorate immunosurveillance (Deng et al., 2014). Against this backdrop of tumor-induced exhaustion, APS and AS-IV emerge as pleiotropic immunomodulators that not only regulate a multifaceted cellular response (Figure 1) but also show potential to synergize with such checkpoint blockade strategies.

Initial preclinical evidence demonstrates that APS supports a CD8^+^ T cell-dominated immune response both through its intrinsic pharmacological activities—namely, increasing the proportion of intra-tumoral cytotoxic CD8^+^ T cells, promoting the generation of effector memory T cells, and enhancing IFN-γ production to establish durable systemic immunity—and by functioning as a potent immune adjuvant within nanoplatforms or combination regimens (Pang et al., 2019; Hwang et al., 2021; Cao et al., 2024). However, the specific direct molecular targets of this complex macromolecule and its precise impact on the breadth of the T-cell receptor (TCR) repertoire have yet to be fully elucidated. Within the metabolically restrictive TME, T cells face severe nutrient deprivation and metabolic competition driven by tumor glycolysis (Paver et al., 2026). Experimental evidence suggests that APS may mitigate T-cell metabolic exhaustion; in murine HCC models, it significantly downregulates the Programmed cell death protein 1 (PD-1^+^) exhausted subset and expands the Cluster of Differentiation 122^+^ C-X-C Chemokine Receptor 3^+^ (CD122^+^CXCR3^+^) memory subpopulation, endowing Chimeric antigen receptor (CAR)-T cells with enhanced persistence (Zhang et al., 2024). Although drawing insights from its ability to inhibit the mammalian target of rapamycin/hypoxia-inducible factor 1-alpha (mTOR/HIF-1α) pathway in murine ulcerative colitis (Liu et al., 2025a) strongly suggests context-dependent metabolic interventions, it is critical to note that these cellular rescues are predominantly observed in well-vascularized rodent models, which may not fully recapitulate the dense, hypoxic stroma of human solid tumors.

Consequently, evaluating whether APS can effectively penetrate the dense stroma and severe hypoxia of advanced human solid tumors to reverse T-cell exhaustion requires further comprehensive evaluation in high-fidelity patient-derived organoid (PDO) models. Beyond direct effects on T cells, preclinical studies indicate that APS modulates the broader immunosuppressive microenvironment by reducing the accumulation of M2-like macrophages (Bamodu et al., 2019) and downregulating PD-L1 expression (Chang H.-L. et al., 2020; He et al., 2022; Wu et al., 2025), thereby attenuating key inhibitory signals that restrict T-cell function. Notably, translating these preclinical observations into rigorously evidence-based immunometabolic interventions will necessitate carefully defining the optimal administration windows—dynamically tracking how the timing of APS administration across distinct stages of tumor evolution ultimately influences therapeutic outcomes.

To overcome the limited efficacy of unformulated active compounds in the face of profound tumor immunosuppression, recent strategies have incorporated AS-IV into engineered nano-delivery systems to enhance cytotoxic T lymphocyte (CTL) function. (Guo et al., 2023; Wang et al., 2024; Wei et al., 2025). In these multifunctional platforms, AS-IV demonstrates a dual utility. First, it can function as a targeting moiety. For example, in the ALFM nanoradiosensitizer developed for preclinical models of triple-negative breast cancer (TNBC), AS-IV utilizes its saponin glycoside structure to bind glucose transporter type 1 (GLUT-1) receptors, thereby mediating targeted transmembrane endocytosis (Wang et al., 2024). Notably, it is crucial to clarify that the activation of the cyclic GMP-AMP synthase - stimulator of interferon genes (cGAS-STING) pathway and subsequent CTL infiltration in this system are primarily driven by degrading Mn^2+^ ions and FePt-mediated reactive oxygen species (ROS) generation, rather than the intrinsic immunomodulatory activity of AS-IV. Furthermore, the reliance on heavy metals (Fe, Pt, Mn) in these carrier systems introduces significant limitations, including complex clearance kinetics and potential long-term systemic toxicity, which necessitate comprehensive evaluation prior to clinical translation (Wang et al., 2024).

Conversely, when functioning as a pharmacological effector, AS-IV has been evaluated as an immunometabolic modulator in complex combination regimens. In preclinical breast cancer and HCC models, it is co-delivered with Oxymatrine (Om) via liposomes (Om-As-Lip) or platelet membrane-cloaked magnetic Metal-Organic Frameworks (MOFs) (PmMN@Om&As) to enhance the efficacy of anti-PD-1 therapy (Guo et al., 2023; Wei et al., 2025). Within these multi-agent strategies, the therapeutic roles are distinctly hypothesized: Om is reported to suppress CAFs, while AS-IV is specifically implicated in promoting the mitochondrial activity of TILs. However, delineating AS-IV’s exact mechanistic contribution presents a nuanced analytical challenge. For instance, in the mice HCC model, the MOF carrier itself proved bioactive by synergistically upregulating the oxygen consumption and proton efflux rates of TILs; therefore, rigorous decoupling through genetic or chemical biology approaches is required to clearly separate the material’s intrinsic effects from AS-IV’s inherent biological targets (Guo et al., 2023). Furthermore, while microfluidic techniques show promise for scaling up co-loaded liposomes (Wei et al., 2025), successfully translating these multifaceted nano-strategies into clinical practice faces significant manufacturing and biosafety hurdles. Addressing these translational limitations—particularly carrier bioactivity, complex pharmacokinetics, and the industrial reproducibility of biomimetic platelet membranes—is essential before such strategies can advance beyond preclinical evaluation (Guo et al., 2023).

**FIGURE 2:**
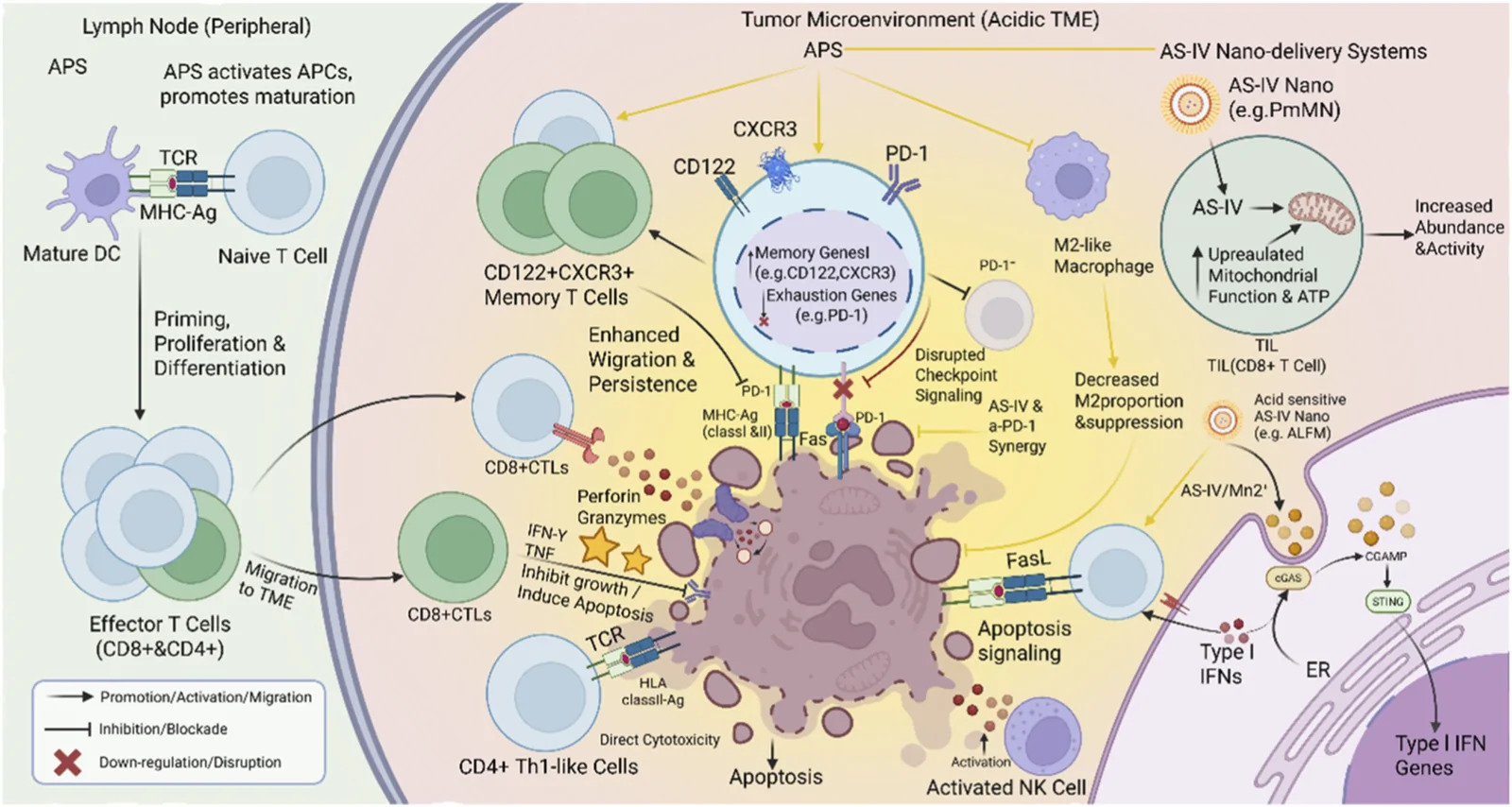
The immunomodulatory network of APS and AS-IV within the tumor microenvironment. Upstream in the peripheral lymph nodes, APS promotes DC maturation to prime naive T cells, facilitating their differentiation and subsequent infiltration into the TME. Within this acidic microenvironment, preclinical models indicate that APS expands the CD122^+^CXCR3^+^ memory T-cell subpopulation, downregulates exhaustion markers (e.g., PD-1), and reduces the accumulation of immunosuppressive pro-repair immune cells. This coordinated cellular response promotes tumor cell apoptosis, mediated by activated CD8^+^ CTLs, CD4^+^ Type 1 T helper (Th1) cells (CD4^+^ Th1 cells), and NK cells via the release of perforin, granzymes, and pro-inflammatory cytokines. Concurrently, AS-IV is utilized within advanced engineered nano-delivery platforms: in these combinatorial systems, PmMN nanocarriers primarily augment mitochondrial Adenosine Triphosphate (ATP) production in TILs, while in acid-responsive ALFM platforms, it is the co-delivered Mn^2+^—rather than AS-IV alone—that triggers the cGAS-STING pathway, inducing Type I interferons (IFNs). Furthermore, experimental evidence suggests that combining AS-IV with anti-PD-1 therapy mitigates inhibitory checkpoint signaling and T-cell exhaustion, thereby supporting sustained anti-tumor immunity.

Taken together, preclinical evidence indicates that APS and AS-IV modulate T-cell immunity within the TME through distinct yet complementary mechanisms (Figure 2). APS appears to primarily facilitate upstream cellular priming and metabolic regulation, whereas AS-IV has been increasingly utilized as an immunometabolic modulator within engineered nano-delivery platforms to enhance targeted interventions. By attenuating key immunosuppressive signals and mitigating effector cell exhaustion in experimental models, both constituents demonstrate potential as promising adjuvants for clinical interventions such as immune checkpoint blockade. Ultimately, translating these preclinical observations into rigorously evidence-based therapies will necessitate meticulously decoupling their intrinsic molecular targets from the synergistic effects of complex nanocarriers, and transitioning validation efforts from predominantly rodent-based models toward high-fidelity, patient-derived systems (Table 1).

**TABLE 1:** Experimental evidence-based summary of immunomodulatory effects of *Astragalus* extracts on T cells in tumors.

Compound/formulation	Immunological effects	Molecular targets/pathways	Tumor models/systems	Level of evidence	Effect attribution	References
Astragalus Polysaccharide (APS)	Promotes the formation and persistence of CD122^+^CXCR3^+^ memory CD8^+^ CAR-T cells; reduces PD-1^+^ exhausted subset; enhances CAR-T cell proliferation and chemotaxis	STAT5 phosphorylation; CXCL9/CXCL10	Mouse HCC xenograft model (Huh7, HepG2); *In vitro* CAR-T cell experiments	*In vivo*/*in vitro*	Intrinsic compound effect	Zhang et al. (2024)
Astragalus Polysaccharide (APS)	Activates CD8^+^ T cells and reverses their inhibitory state; decreases LAG3 expression in CD8^+^ T cells	STAT3/Galectin-3/LAG3 pathway	AOM/DSS inflammation-induced CRC mouse model; MC38 and CD8^+^ T cells co-culture *in vitro*	*In vivo*/*in vitro*	Intrinsic compound effect	Li Q. et al. (2024)
PLGA-Astragalus Polysaccharide (NP-TCL@APS)	Promotes preferential uptake and activation by DCs; enhances antigen-specific CD8^+^ T cell response; inhibits tumor growth	DC maturation (specific pathway unspecified)	Mouse CRC tumor-bearing model; *In vitro* DC uptake experiment	*In vivo*/*in vitro*	Nanoplatform effect (PLGA carrier + APS + TCL)	Cao et al. (2024)
Astragalus Polysaccharide (APS)	Intranasal administration: increases DC number and activation in mLN; activates NK and T cells; enhances anti-PD-L1 efficacy (combination inhibits B16 lung metastasis)	CCR7 upregulation	Mouse B16 lung metastatic melanoma model	*In vivo*	Intrinsic compound effect	Hwang et al. (2021)
Astragalus Polysaccharide (APS)	Promotes CD4^+^ and CD8^+^ T cell proliferation; reduces PD-1^+^ CD4^+^ and PD-1^+^ CD8^+^ T cells; attenuates PD-L1 expression; initiates prostate cancer cell apoptosis	PD-1/PD-L1 interaction	Prostate cancer cells (PC-3) and macrophages/T cells *in vitro*	*In vitro*	Intrinsic compound effect	Wu et al. (2025)
Honey-processed Astragalus Polysaccharide (HP-APS)	Induces tumor cell apoptosis (G1 to S phase arrest); increases HSP70, CRT, MHC-I, CD80/CD86, and ATP release; significantly improves CD8^+^ T cell proliferation and infiltration; increases CD4/CD8 ratio	ICD molecules (CRT, HSP70, MHC-I, CD80/CD86); ATP release	Human A549, mouse MC38 and B16 cell lines *in* *vitro*; Mouse B16 melanoma model *in vivo*	*In vivo*/*in vitro*	Intrinsic compound effect	Sha et al. (2022)
Astragalus Polysaccharide (APS)/scFv S12 antibody	APS induces specific anti-PD-1 antibodies; isolated scFv S12 blocks PD-1/PD-L1 interaction, reverses T cell exhaustion, reduces TAMs in tumor tissue	PD-1/PD-L1 interaction	PBMCs *in vitro*; Animal tumor model	*In vivo*/*in vitro*	Antibody derivative effect (APS-stimulated specific antibody)	Chang F.-L. et al. (2020)
Astragaloside IV (AS-IV) + anti-PD-1	Significantly reduces tumor volume and weight in LLC-bearing mice; enhances tumor cell apoptosis and suppresses proliferation; increases M1 macrophage markers (mCD86), reduces M2 markers (mCD206); upregulates T cell activation markers (mCD69)	Inactivation of PI3K/Akt and ERK signaling pathways	Mouse Lewis lung carcinoma (LLC) subcutaneous model	*In vivo*	Combination therapy effect (AS-IV + anti-PD-1)	Wu T. et al. (2024)
Astragaloside IV (AS-IV) + anti-PD-1	Dose-dependently inhibits CT26 cell proliferation and induces apoptosis; reduces M2 and increases M1 macrophages; decreases TGF-β, IL-10, VEGF-A, increases IFN-γ, IL-12, TNF-α; combination exhibits synergistic anti-tumor effects and increases T cell infiltration	M2 to M1 macrophage repolarization (specific pathway unspecified)	Mouse CRC subcutaneous model (CT26); Bone marrow-derived macrophages *in vitro* polarization	*In vivo*/*in vitro*	Combination therapy effect (AS-IV + anti-PD-1)	Liu et al. (2020)
Astragaloside IV (AS-IV)	Blocks IDO1 and GBP1 interaction, reduces extracellular secretion of IDO1, alleviates T cell exhaustion, inhibits tumor progression	IDO1/GBP1 protein interaction	Human lung cancer cell lines *in vitro*; Mouse lung cancer model *in vivo*	*In vivo*/*in vitro*	Intrinsic compound effect	Meng et al. (2020)
Astragaloside III (As) + Ce6 ((As + Ce6)@MSNs-PEG)	Activates NK cells and CD8^+^ T cells; enhances immune cell infiltration; increases cytotoxicity of NK cells and CD8^+^ T cells	T-bet transcription factor; IFN secretion	Colon cancer cells *in vitro*; Tumor-bearing mice *in vivo*	*In vivo*/*in* *vitro*	Nanoplatform effect (MSNs-PEG carrier + As + Ce6 PDT)	Wu et al. (2021)
Astragaloside IV (AS-IV)	Suppresses tumor growth; downregulates regulatory T cells (Tregs) and upregulates cytotoxic T lymphocytes (CTLs)	IDO expression inhibition	IDO-overexpressed murine Lewis lung carcinoma orthotopic model	*In vivo*/*in vitro*	Intrinsic compound effect	Zhang et al. (2014)
Oxymatrine (Om) + Astragaloside IV (As)	Om suppresses CAF activation (downregulates FAP and α-SMA); As improves TIL mitochondrial activity (increases mitochondria and cristae quantity); optimal ratio (2:1) enhances CD4^+^ and CD8^+^ T cell infiltration and tumor apoptosis	FAP/α-SMA (CAF); Mitochondrial morphology (TILs)	Trans-CAF system *in vitro*; TNBC 4T1-luc *in situ* mouse model *in vivo*	*In vivo*/*in vitro*	Combination therapy effect (Om + As)	Wang H. et al. (2023)
Om-As-Lip (Co-loaded liposomes)	Enhances anti-tumor efficacy of α-PD-1; inhibits CAF activation and boosts TIL activity	CAF activation; TIL mitochondrial activity	Breast cancer mouse model	*In vivo*	Nanoplatform effect (Liposome carrier + Om + As)	Wei et al. (2025)
PmMN@Om&As (Magnetic MOF nanoplatform)	Targets HCC tissue; inhibits CAFs; increases TIL levels and activity; upregulates TIL mitochondrial function	TIL mitochondrial function (oxygen consumption rate and proton efflux rate)	HCC microenvironment	*In vivo*/*in vitro*	Nanoplatform effect (MOF carrier + Platelet membrane + Om + As)	Guo et al. (2023)

##### 3.1.1.2 Promotion of dendritic cell maturation and antigen presentation

As professional Antigen-presenting cells (APCs), DCs capture tumor-associated antigens (TAAs) in an immature state (Böttcher and Reise Sousa, 2018; Fu and Jiang, 2018; Patente et al., 2018; Tiberio et al., 2018) and undergo profound phenotypic reprogramming upon recognizing damage-associated molecular patterns (DAMPs) via pattern-recognition receptors (PRRs) like Toll-like receptors (TLRs) (Zelenay and Reise Sousa, 2013; Constantino et al., 2017). This maturation involves upregulating major histocompatibility complex class II (MHC-II), co-stimulatory molecules Cluster of Differentiation 80/86 (CD80/CD86), and the chemokine receptor C-C Chemokine Receptor 7 (CCR7) to secrete Interleukin-12 (IL-12) and initiate T-cell responses in draining lymph nodes (dLNs) (Byrne and Halliday, 2002; Patente et al., 2019). Crucially, this activation depends on a rapid glycolytic surge (O’Neill and Pearce, 2016) and is theoretically driven by immunogenic cell death (ICD)-associated cytokines including Interleukin-1β (IL-1β), Interleukin-6 (IL-6), and TNF-α (Gardner and Ruffell, 2016). However, the immunosuppressive TME frequently restricts this process; tumors may exploit the Cluster of Differentiation 47 (CD47)-signal regulatory protein alpha (SIRPα) axis to impede phagocytosis (Xu et al., 2017), while severe glucose depletion limits the essential glycolytic burst, often driving DCs toward a tolerogenic state (O’Neill and Pearce, 2016). Consequently, achieving robust antigen presentation generally requires concurrent adjuvant interventions to support DC metabolic fitness and overcome these inhibitory barriers.

Within the immunosuppressive TME, APS functions as an immunomodulator capable of mitigating DC dysfunction. To facilitate the delivery of tumor antigens to the dLNs, APS is frequently incorporated into engineered carrier systems, such as poly (lactic-co-glycolic acid) (PLGA) nanoparticles co-loaded with tumor cell lysates (TCL) (NP-TCL@APS) (Cao et al., 2024), or pH-responsive, calcium phosphate-wrapped tumor nanovaccines (APS-NVs) (Xu et al., 2022; Li N. et al., 2024). Notably, while these nanoplatforms successfully upregulate MHC-II and CD80/CD86 to induce Th1-biased or tumor-specific CD8^+^ T-cell responses in preclinical models (Xu et al., 2022; Cao et al., 2024; Li N. et al., 2024), their reliance on crude TCLs or highly immunogenic model antigens may not fully reflect the heterogeneous neoantigen landscape of spontaneous human solid tumors. Furthermore, evaluating these delivery systems highlights the necessity to methodologically decouple APS’s intrinsic adjuvant properties from the non-specific immunogenic effects of the synthetic polymers.

Beyond nanoparticle-mediated delivery, experimental evidence indicates that APS acts directly via intercellular crosstalk. Although APS has been shown to upregulate Blood Dendritic Cell Antigen 1/3 (BDCA1/BDCA3) markers on human peripheral blood dendritic cells (PBDCs) and promote autologous T-cell proliferation (An et al., 2022), it is important to note that this *ex vivo* activation occurs in controlled environments. Such settings may not fully recapitulate the severe metabolic limitations and suppressive cytokine profiles characteristic of the *in vivo* TME.

Finally, in combinatorial treatment strategies, APS demonstrates synergistic potential. For instance, in preclinical non-small cell lung cancer (NSCLC) xenograft models, APS administration attenuates the immunosuppressive microenvironment by reducing M2-like macrophage populations, which consequently enhances cisplatin efficacy and alleviates chemotherapy-associated cachexia (Bamodu et al., 2019). Concurrently, the intranasal co-administration of APS with an anti-PD-L1 antibody in murine models of melanoma lung metastasis enhances mucosal immunity by activating NK cells and CD8^+^ T cells (Hwang et al., 2021). However, determining whether this mucosal priming possesses the penetrative therapeutic efficacy required to eradicate deep-seated, stroma-dense primary tumors remains a critical objective for future clinical validation.

Furthermore, AS-IV appears to exert bidirectional, context-dependent modulatory effects by differentially regulating DC phenotypes (Yang et al., 2020; Guo H. et al., 2022). In experimental models related to HCC, AS-IV demonstrates immune-stimulating potential. Notably, it markedly upregulates DC maturation markers—including Cluster of Differentiation 40 (CD40), CD80, Cluster of Differentiation 83 (CD83), CD86, and Human Leukocyte Antigen-DR (HLA-DR)—and enhances IL-12 secretion to improve T cell tumor-killing efficiency *in vitro* (Guo H. et al., 2022). However, interpreting this activation requires careful consideration of the experimental design. Because this *ex vivo* HCC study utilized DCs derived from the peripheral blood of healthy donors rather than tumor-infiltrating DCs isolated from the actual TME (Guo H. et al., 2022), the ultimate capacity of AS-IV to effectively reverse established intra-tumoral tolerance requires comprehensive evaluation in physiologically complex models that accurately recapitulate the severe immunosuppressive imprinting of human solid tumors.

Conversely, in autoimmune/inflammatory models such as experimental autoimmune encephalomyelitis (EAE), AS-IV seemingly transitions into an immunosuppressive role to mitigate autoreactive immune surveillance. Intriguingly, early administration attenuates DC maturation by reducing Cluster of Differentiation 11c (CD11c), CD86, CD40, and MHC-II expression—alongside decreased IL-6 and IL-12 secretion—to impede CD4^+^ T cell differentiation into T Helper 1/17 Cells (Th1/Th17) via the inhibition of Nuclear Factor-Kappa B (NF-κB) and Mitogen-Activated Protein Kinase (MAPK) signaling pathways (Yang et al., 2020). This bidirectional regulatory capability is likely shaped by the compound’s interaction with differing baseline inflammatory milieus rather than a solitary molecular switch. Thus, defining the precise systemic outcomes of this context-dependent modulation warrants further validation through cell-type-specific genetic models. Taken together, these observations suggest that APS and AS-IV act as versatile DC-centric immunomodulators; however, their intrinsic capacities must be carefully contextualized. Maximizing their clinical potential will likely require integrating these context-dependent phenotypic effects with engineered delivery platforms to amplify T-cell-directed antitumor immunity within the TIME.

Collectively, preclinical evidence suggests that APS and AS-IV modulate the dendritic cell network within the TIME through distinct mechanisms. APS primarily enhances antigen cross-presentation and systemic Th1/CD8^+^ priming when utilized as an immunological adjuvant within engineered nanovaccines, while AS-IV exhibits bidirectional, context-dependent regulatory effects, capable of either enhancing tumor-directed immune stimulation or mediating inflammatory dampening depending on the experimental model. By mitigating microenvironment-induced DC dysfunction and facilitating the transition from innate antigen recognition to adaptive T-cell responses, these constituents demonstrate promising utility as synergistic adjuvants for current immunotherapies; however, extensive clinical validation remains necessary to confirm these preclinical observations (Table 2).

**TABLE 2:** Experimental evidence-based summary of immunomodulatory effects of *Astragalus* extracts on dendritic cells in tumor and adjuvant settings.

Compound/formulation	Immunological effects	Molecular targets/pathways	Tumor models/systems	Level of evidence	Effect attribution	References
Astragalus Polysaccharide (AMP)	Induces morphological changes and upregulates activation markers in human monocyte-derived DCs (MDDCs); activates BDCA1^+^ and BDCA3^+^ PBDCs; promotes autologous T cell proliferation and activation	Upregulation of DC activation markers; Inflammatory cytokine secretion	Human peripheral blood-derived DCs (PBDCs) and monocyte-derived DCs (MDDCs)	*Ex vivo*	Intrinsic compound effect	An et al. (2022)
Astragalus Polysaccharide (PG2)	Promotes functional maturation of DCs, enhancing T cell-mediated anti-cancer immunity; increases M1/M2 macrophage polarization ratio in NSCLC; downregulates IL-6 and IL-10; synergistically enhances cisplatin efficacy and alleviates cachexia	M1/M2 polarization regulation; IL-6/IL-10 downregulation	NSCLC H441/H1299 cells *in vitro*; NSCLC patient clinical samples *ex vivo*; NSCLC mouse xenograft model *in vivo*	*In vitro*/*ex vivo*/*in vivo*	Intrinsic compound effect	Bamodu et al. (2019)
Astragalus mongholicus Polysaccharides (ASP)	Increases co-expression of CD11c and MHC-II on murine bone marrow-derived DCs (optimal at 100 μg/mL); enhances IL-12 secretion; promotes morphological maturation of DCs (long protrusions)	CD11c/MHC-II upregulation; IL-12 secretion	Murine bone marrow-derived DCs (BMDCs) cultured *in vitro*	*In vitro*	Intrinsic compound effect	Shao et al. (2006)
Astragalus Polysaccharide Liposome (APSL)	Improves macrophage phagocytosis, IL-6/IL-12 content, and NO/iNOS secretion; promotes DC precursor proliferation; enhances DC capabilities in stimulating T cell proliferation and antigen presentation; upregulates IFN-γ, IL-2, CD80, and CD86 in DCs	iNOS/NO pathway; Cytokine (IL-6, IL-12, IFN-γ, IL-2) secretion; CD80/CD86 upregulation	Murine peritoneal macrophages and bone marrow-derived DCs	*In vitro*	Nanoplatform effect (Liposome carrier + APS)	Zhang et al. (2017)
Astragalus Polysaccharide (APS)	Induces DC dendrite elongation, decreases phagocytic ability, and causes phenotypic changes (upregulation of CD40/MHC-II/CD80/CD86); increases NO levels	NO-mediated pathway	Murine bone marrow-derived DCs	*In vitro*	Intrinsic compound effect	Zhu N. et al. (2016)
OVA/APS Integrated Nanocomplexes (APS-NVs)	Wrapped with sheddable calcium phosphate; efficiently taken up by DCs; stimulates DC maturation; improves antigen cross-presentation efficiency; activates cytotoxic T cells; achieves better anti-tumor effect in B16-OVA melanoma than OVA + Alum	Dectin-1 and Toll-like receptors 2 and 4 (TLR2/4)	Murine B16-OVA melanoma model *in vivo*; DCs *in vitro*	*In vivo*/*in vitro*	Nanoplatform effect (Calcium phosphate wrapper + APS + OVA)	Li et al. (2024)
PLGA-Astragalus Polysaccharide (NP-TCL@APS)	Preferentially uptaken by DCs; promotes DC activation and maturation; enhances antigen-specific CD8^+^ T cell response; inhibits CRC tumor growth with good biocompatibility	DC activation (specific pathway unspecified)	Mouse CRC tumor-bearing model; *In vitro* DC uptake and activation	*In vivo*/*in vitro*	Nanoplatform effect (PLGA carrier + APS + TCL)	Cao et al. (2024)
CD-205-Targeted Liposomes (iLPSM) with APS/OVA	Promotes macrophage proliferation and rapid phagocytosis; significantly improves OVA-specific IgG/IgG isotype titers; promotes T and B lymphocyte differentiation and DC maturation; prolongs nanoparticle retention time at the injection site	CD-205 receptor-mediated DC targeting	Murine immune model *in vivo*; Macrophage phagocytosis *in vitro*	*In vivo*/*in vitro*	Nanoplatform effect (CD-205 targeted liposome + APS + OVA)	Liu et al. (2023)
pH-Responsive APS-encapsulated PLGA Nanoparticles (OVA-loaded pH-responsive APSPs)	pH-responsive release (acidic environment); enhances MHC-II, CD80, CD86 expression and macrophage phagocytic ability; stimulates Th1-biased immune response; promotes splenic lymphocyte and DC proliferation, differentiation, and maturation; enhances specific IgG, IL-4, IL-6, IFN-γ, and TNF-α	MHC-II/CD80/CD86 upregulation; Th1-biased immune response	Splenic lymphocytes, DCs, and macrophages *in vitro*; Mouse immune model *in vivo*	*In vivo*/*in vitro*	Nanoplatform effect (pH-responsive PLGA + APS + OVA)	Xu et al. (2022)
APS/AuNR/PLGA-PEG Nanoparticles	Synergistic agent for Focused Ultrasound (FUS); upregulates MHC-II, CD80, CD86 on DCs; decreases DC phagocytosis and enhances IL-12/IFN-γ production; boosts TNF-α, IFN-γ, IL-4, IL-10, and IgG1 *in vivo*	FUS-induced immune boosting; Cytokine production	Rabbit liver FUS model *in vivo*; DCs co-culture *in vitro*	*In vivo*/*in vitro*	Nanoplatform effect (FUS + AuNRs + PLGA-PEG + APS)	Xiong et al. (2020)
Astragalus membranaceus (Am) + Codonopsis pilosulae (Cp) Polysaccharides	*Ex vivo* DC vaccine approach: enhances CD4^+^ and CD8^+^ T cell proliferation; exhibits strong anti-4T1 metastasis activity; augments CD40, CD80, CD86 on DCs; activates specific immune functions via cytokine/chemokine mediation	Cytokine/chemokine mediation network	4T1 mammary carcinoma metastasis mouse model *in vivo*; DCs *in vitro*	*In vivo*/*in vitro*	Combination formulation effect (Am + Cp)	Chang et al. (2015)
Astragaloside IV (ASI)	Acts as an immunopotentiator during DC induction; significantly upregulates CD14, CD40, CD80, CD83, CD86, and HLA-DR; increases IL-12 secretion; enhances sensitized DC-stimulated T lymphocyte proliferation; increases specific killing rate of CTLs against SMMC-7721 hepatoma cells	IL-12 secretion; Upregulation of DC surface markers (CD40/CD80/CD83/CD86/HLA-DR)	Human peripheral blood-derived DCs *in vitro*; SMMC-7721 hepatoma cell killing assay	*In vitro*	Intrinsic compound effect	Guo H. et al. (2022)

##### 3.1.1.3 Augmentation of natural killer cell cytotoxicity

As a heterogeneous innate immune component (Wu et al., 2020), NK cells inhibit tumor progression via perforin/granzyme-dependent cytotoxicity (Chen and Hambardzumyan, 2018; Chiossone et al., 2018), shape adaptive immunity by secreting cytokines like IFN-γ, TNF-α, and Interleukin-10 (IL-10) (Vivier et al., 2011; Wu et al., 2020), and preferentially target major histocompatibility complex class I (MHC-I) deficient tumor cells (Amin and Shankar, 2015; Wu et al., 2020). However, this immunosurveillance is frequently compromised by the TME, where inhibitory receptor signaling (e.g., PD-1/PD-L1) and metabolic stressors—including hypoxia, nutrient deprivation, and tumor-derived lactate—collectively attenuate NK cell function (Concha-Benavente et al., 2018; Terrén et al., 2019; Fischer et al., 2007). Consequently, optimizing the therapeutic potential of NK cells necessitates targeted interventions that can simultaneously amplify their inherent cytotoxicity and mitigate these synergistic constraints.

Recent preclinical studies reveal that diverse APS formulations can modulate NK cell activity and anti-tumor immune responses, a process fundamentally dictated by a complex structure-activity relationship (SAR) (Li et al., 2014; Yu et al., 2018; Li C. et al., 2020). For instance, *in vitro* studies demonstrate that the water-soluble APS fraction relies on essential sulfate motifs to stimulate nitric oxide (NO) production in murine macrophages and drive robust NK cell proliferation and cytotoxicity against the HeLa Cervical Carcinoma Cell Line (Li C. et al., 2020). Although this sulfated fraction promotes the release of IFN-γ, granzyme B, and perforin—and has been suggested to facilitate target recognition by upregulating molecules such as MHC-I chain-related molecules A (MICA) and B (MICB) in specific cellular models (Li C. et al., 2020)—the precise receptor-ligand interactions distinguishing this direct NK cell activation from secondary, cytokine-driven responses merit further rigorous characterization.

In addition to structural modifications like sulfation, the overarching chemical makeup of APS plays a critical role in shaping its immune-regulating properties. Notably, in murine models of HCC and sarcoma, selenium-containing Astragalus polysaccharide (Se-PAM) has been shown to reduce the accumulation of immunosuppressive M2-phenotype tumor-associated macrophages (TAMs) while enhancing NK and CD8^+^ T cell activity (Li et al., 2014). Similarly, alcohol-soluble Astragalus polysaccharide (ASP) elevates systemic levels of TNF-α, Interleukin-2 (IL-2), and IFN-γ to modulate the activity of macrophages, lymphocytes, and NK cells *in vivo* (Yu et al., 2018). Despite these promising observations, the robust systemic pro-inflammatory cascades stimulated by these pleiotropic macromolecules demand careful pharmacological evaluation. Specifically, accurately defining their pharmacokinetic distribution and ensuring strict spatial confinement within the TME will be essential to maximize therapeutic efficacy while mitigating potential off-target toxicities.

Finally, intranasal administration of APS can serve as an effective mucosal adjuvant, successfully recruiting DCs to boost NK and CD8^+^ T-cell responses and augment anti-PD-L1 therapy in murine models of metastatic melanoma (Hwang et al., 2021). Despite these promising results, advancing these distinctly sulfated, selenium-enriched, and alcohol-soluble fractions beyond current murine models will require innovative targeted delivery strategies to ensure successful penetration of advanced human solid tumors.

Demonstrating versatile immunomodulatory properties, AS-IV enhances NK cell defenses not only through direct molecular interactions but also as an integral component of complex formulations. *In vitro*, advanced two-dimensional NK-92MI cell membrane chromatography coupled with mass spectrometry has identified AS-IV—alongside isoastragaloside I—as a ligand that directly binds to NK membrane targets, dose-dependently enhancing NK cell-mediated cytotoxicity against the K562 chronic myeloid leukemia cell line (Chai et al., 2022). Concurrently, in more complex multi-agent regimens, AS-IV is a primary component of formulations like the ginseng-astragalus-oxymatrine (GAO) injection. In murine models of cyclophosphamide-induced immunosuppression, GAO administration ameliorates immune dysfunction via the NF-κB signaling pathway; it elevates NK cell cytotoxic activity while simultaneously stimulating macrophage phagocytosis, T and B cell immunity, and the secretion of pro-inflammatory cytokines (TNF-α, IL-6) (Li Y. et al., 2021).

Consequently, a clear mechanistic distinction emerges from these preclinical models: while APS primarily modulates systemic multicellular networks to ameliorate the immunosuppressive microenvironment, AS-IV exhibits a propensity for direct NK target engagement alongside its combinatorial signaling role. However, transitioning these insights toward clinical application requires addressing significant methodological limitations. Specifically, validating AS-IV’s putative direct membrane targets in physiologically complex *in vivo* models—rather than relying solely on immortalized cell lines (Chai et al., 2022)—and rigorously decoupling its precise pharmacological contribution from the synergistic bioactive matrix of GAO (Li Y. et al., 2021), represent crucial analytical objectives for rationally evaluating these combinatorial immunotherapies.

In summary, current experimental models indicate that APS and AS-IV support NK cell immunosurveillance through distinct but complementary mechanisms: APS utilizes specific structural formulations to modulate broader multicellular networks, while AS-IV demonstrates a capacity for direct engagement with NK cell receptors. By enhancing NK cell cytotoxicity and attenuating immunosuppressive barriers within the TME, both compounds show promise in experimental models as potential adjuvants to augment the efficacy of immune checkpoint therapies (Table 3).

**TABLE 3:** Experimental evidence-based summary of immunomodulatory effects of *Astragalus* extracts on natural killer cells in tumors and *in vitro* studies.

Compound/formulation	Immunological effects	Molecular targets/pathways	Tumor models/systems	Level of evidence	Effect attribution	References
Astragalus Polysaccharide (APS)	Intranasal administration: increases the number and activation of lineage^−^CD11c^+^ DCs in mesenteric lymph nodes; activates NK and T cells; APS/anti-PD-L1 combination inhibits B16 melanoma lung metastasis; anti-cancer effect is mitigated after depletion of NK and CD8^+^ T cells	CCR7 upregulation (mediating DC migration)	Mouse B16 lung metastatic melanoma model; NK/CD8^+^ T cell depletion assays	*In vivo*	Intrinsic compound effect (acting as a mucosal adjuvant)	Hwang et al. (2021)
Selenium-containing Astragalus Polysaccharides (PAMs)	Selenium-dependently inhibits the *in vivo* proliferation of H22 ascitic hepatoma and S180 sarcoma; ameliorates CD4^+^ T cell apoptosis and serum cytokine dysregulation; enhances cytotoxic activities of NK and CD8^+^ T cells; improves macrophage phagocytosis and suppresses M2-like TAM polarization	Selenium-dependent immunomodulation (selenium content varies significantly among PAMs from different habitats)	Mouse H22 ascitic hepatoma model; Mouse S180 sarcoma model	*In vivo*	Intrinsic compound effect (immunomodulatory activity is highly dependent on selenium content)	Li et al. (2014)
Alcohol-soluble Astragalus Polysaccharide (ASP)	Significantly inhibits the *in vivo* growth of H22 hepatoma cells; increases serum levels of TNF-α, IL-2, and IFN-γ; enhances the activities of macrophages, lymphocytes, and NK cells; induces tumor cell apoptosis	Elevation of serum cytokines (TNF-α, IL-2, IFN-γ); Immune cell activation	Mouse H22 hepatoma model	*In vivo*	Intrinsic compound effect (Neutral polysaccharide, ∼2.1 × 10^3^ Da, composed of arabinose, galactose, glucose, and mannose)	Yu et al. (2018)
Water-soluble Astragalus membranaceus Polysaccharide (AMP)	AMP and its derivatives (deproteinated and desulfated AMP) significantly increase NK cell proliferation (113.1%–128.7%) and cytotoxicity against HeLa cells (37.4%–55.5%); Desulfation significantly attenuates NK cell activation (decreases IFN-γ, TNF-α, Granzyme-B, and NKp44) and RAW264.7 NO production	NF-κB and MAPKs pathways; Sulfate groups play a crucial role in activation	RAW264.7 murine macrophages and NK cells *in vitro*	*In vitro*	Intrinsic compound effect (dependent on structural modification/sulfate groups)	Li C. et al. (2020)
Astragalus Polysaccharide (APS) + Radiotherapy	Upregulates MICA and MICB expression on the tumor cell surface; activates NK cells via NKG2D receptor binding; increases ERK phosphorylation in NK cells; promotes the release of IFN-γ, Granzyme B, and Perforin, increasing tumor sensitivity to radiotherapy	MICA/B-NKG2D axis; ERK signaling pathway in NK cells	Mouse H22 hepatocellular carcinoma model (radiotherapy combined)	*In vivo*	Combination therapy effect (APS + Radiotherapy)	Mu et al. (2019)
Isoastragaloside I and Astragaloside IV (AS-IV)	Screened via a 2D NK-92MI CMC system; dose-dependently promotes the specific killing effect of NK cells on K562 cells	Enhances NK cell cytotoxicity (specific pathway unspecified)	K562 cells and NK-92MI cells *in vitro*	*In vitro*	Intrinsic compound effect	Chai et al. (2022)
Ginseng-Astragalus-oxymatrine (GAO)	Significantly improves nonspecific immunity (increases thymus/spleen indices, peripheral leukocytes, TNF-α, IL-6); enhances macrophage phagocytosis and NK cell cytotoxic activity; restores cellular and humoral immunity in immunosuppressed mice	Activation of ROS and NF-κB signaling pathways	CTX-induced immunosuppressive mouse model; RAW 264.7 cells *in vitro*	*In vivo*/*in vitro*	Combination formulation effect (Ginseng + Astragalus + Oxymatrine)	Li Y. et al., (2021)

##### 3.1.1.4 Induction of anti-tumorigenic M1 macrophage polarization

Upon infiltrating the TME, macrophages frequently dominate the leukocyte landscape as TAMs (Murray et al., 2014). While physiologically responsible for maintaining tissue homeostasis and systemic immune defense (Varol et al., 2015), TAMs exhibit profound functional plasticity conventionally conceptualized along an M1–M2 continuum (Zhang and Sioud, 2023). When stimulated by specific cytokines—including Granulocyte-Macrophage Colony-Stimulating Factor (GM-CSF), IL-12, Interleukin-18 (IL-18), IFN-γ, and TNF-α (Zhu J. et al., 2016; Goswami et al., 2021)—macrophages can polarize toward a tumoricidal M1 phenotype characterized by robust phagocytic activity, upregulated major histocompatibility complex (MHC) expression, and the secretion of pro-inflammatory mediators (such as IL-1β, IL-6, and TNF-α) to facilitate malignant cell elimination (Lewis and Pollard, 2006; Porta et al., 2015; Schultze and Schmidt, 2015; Ngambenjawong et al., 2017). However, within the physiological complexity of the true TME, TAMs rarely exist in absolute binary states; instead, they frequently adopt intermediate phenotypes dynamically shaped by local metabolic constraints. Consequently, advancing from this foundational continuum toward a more high-resolution, context-dependent understanding of macrophage plasticity represents an important objective for rationally optimizing targeted immunotherapies.

As an immunomodulator in the TME, APS facilitates the phenotypic shift of macrophages from a pro-tumorigenic state to an anti-tumorigenic M1 profile. At the molecular level, APS stimulates macrophage-mediated cytotoxicity. In murine breast cancer models, APS activates the NOTCH signaling pathway to upregulate crucial M1 markers—including inducible nitric oxide synthase (iNOS), IL-6, TNF-α, and C-X-C motif chemokine 10 (CXCL10)—thereby effectively inhibiting 4T1 tumor growth (Wei et al., 2019). Similarly, *in vitro* co-culture studies demonstrate that this M1 activation triggers the secretion of NO and TNF-α to suppress the growth of the MCF-7 human breast cancer cell line. This induces apoptosis, evidenced by a 13.26-fold increase in the Bax/Bcl-2 ratio (Li et al., 2019). Notably, these mechanistic insights predominantly rely on immortalized murine cell lines, such as RAW264.7 (Li et al., 2019; Wei et al., 2019). Consequently, whether these *in vitro* responses fully capture the gradient-driven plasticity of primary human TAMs within a metabolically restrictive TME remains an important area for future validation.

Beyond *in vitro* observations, APS administration has been shown to modulate the M1/M2 balance *in vivo*. In preclinical NSCLC models, it enhances cisplatin efficacy by downregulating pro-tumorigenic IL-6 and IL-10 while concurrently reducing the M2 population (Bamodu et al., 2019). Furthermore, APS has been incorporated as a structural component in advanced hybrid nanoparticles (HP-NPs@PTX/Bai) designed to target TAMs. This platform facilitates the reprogramming of the M2 phenotype toward M1 while co-delivering chemotherapeutics like paclitaxel and baicalin (Guo C. et al., 2022). Intriguingly, although this nanodelivery strategy synergistically remodels the TME and mitigates systemic toxicity, it introduces a distinct analytical challenge. Because baicalin itself possesses TAM-re-educating properties, rigorously decoupling the intrinsic immunomodulatory properties of the APS structural matrix from the pharmacological effects of the co-delivered agents will require carefully designed, patient-derived models to rationally optimize these interventions (Guo C. et al., 2022).

In parallel, AS-IV acts as a distinct pharmacological driver of macrophage plasticity, effectively re-educating pro-tumorigenic M2 macrophages toward the anti-tumorigenic M1 phenotype. In murine CRC liver metastasis models, AS-IV has been shown to disrupt intercellular tumor-macrophage communication. It downregulates Neutral Sphingomyelinase 2/Ras-Related Protein Rab27a (nSMase2/Rab27a) expression to inhibit the release of tumor-derived extracellular vesicles (EVs). This blockade effectively restrains the activation of M2-type TAMs in the TME (Zhou et al., 2024). Concurrently, AS-IV promotes M1 macrophage polarization in murine CRC models, thereby altering the cytokine balance. It significantly reduces anti-inflammatory factors like Transforming Growth Factor-β (TGF-β), IL-10, and Vascular Endothelial Growth Factor-A (VEGF-A), while simultaneously upregulating pro-inflammatory mediators, including IFN-γ, IL-12, and TNF-α (Liu et al., 2020).

Furthermore, AS-IV exhibits synergistic potential when combined with immune checkpoint blockade. In both lung cancer (LC) and CRC preclinical models, co-administration with an anti-PD-1 antibody amplifies anti-tumor responses by enhancing M1 polarization and T-cell infiltration (Liu et al., 2020; Wu T. et al., 2024). While AS-IV combined with anti-PD-1 antibodies has demonstrated promising combinatorial effects in LC models by inactivating Phosphatidylinositol 3-Kinase/Protein Kinase B (PI3K/Akt) and Extracellular Signal-Regulated Kinase (ERK) signaling pathways (Wu T. et al., 2024), a distinct analytical challenge remains. Intriguingly, it remains mechanistically ambiguous whether this M1 polarization is driven by AS-IV’s direct engagement with macrophage surface receptors, or if it merely represents a secondary consequence of reduced tumor burden and altered EV signaling (Liu et al., 2020; Zhou et al., 2024). Consequently, rigorously decoupling these precise mechanisms will be essential for future research. By clarifying these pathways and evaluating advanced nanodelivery platforms to improve pharmacologic reach, these Astragalus constituents can be systematically optimized to modulate macrophage plasticity and support anti-tumor immunity.

Taken together, existing preclinical data demonstrate that APS and AS-IV employ divergent yet synergistic pharmacological strategies to reprogram TAMS. APS systematically alters the M1/M2 balance to promote macrophage-mediated cytotoxicity, while AS-IV disrupts intercellular tumor signaling to drive M1 repolarization. By re-educating pro-tumorigenic M2 macrophages toward anti-tumorigenic M1 phenotypes, both compounds demonstrate potential in experimental models to augment the efficacy of chemotherapies and immune checkpoint blockades. However, advancing these interventions toward clinical application requires rigorously decoupling their direct pharmacological targets from secondary microenvironmental changes utilizing high-fidelity, patient-derived models (Table 4).

**TABLE 4:** Experimental evidence-based summary of immunomodulatory effects of *Astragalus* extracts on macrophages/M1 polarization in tumors.

Compound/formulation	Immunological effects	Molecular targets/pathways	Tumor models/systems	Level of evidence	Effect attribution	References
Astragalus Polysaccharide (APS)	Promotes macrophage-mediated tumor cell apoptosis and G2-phase cell cycle arrest; increases thymus and spleen indices; enhances splenic lymphocyte proliferation and peritoneal macrophage phagocytosis; upregulates peripheral blood IL-2, TNF-α, and IFN-γ; synergizes with 5-FU to enhance anti-tumor effects and alleviate chemotherapy-induced immunosuppression	Mitochondrial apoptosis pathway; Upregulation of IL-2/TNF-α/IFN-γ	Mouse 4T1 breast cancer model; *In vitro* macrophage-4T1 co-culture	*In vivo*/*in vitro*	Intrinsic compound effect	Li W. et al. (2020)
Astragalus Polysaccharides (APS)	Increases macrophage secretion of NO, TNF-α, IL-1β, and IL-6; enhances immune organ indices and tumor apoptosis rate; decreases tumor weight; effects are abolished in TLR4- or MyD88-deficient mice	TLR4-MyD88 dependent signaling pathway (TLR4, MyD88, TRAF-6, NF-κB, AP-1)	Mouse EAC tumor-bearing model (TLR4 WT/deficient; MyD88 WT/deficient)	*In vivo*/*in vitro*	Intrinsic compound effect	Zhou et al. (2017)
Astragalus Polysaccharide (APS)	Activates macrophages to inhibit breast cancer cell proliferation; induces G1-phase cell cycle arrest; upregulates Bax/Bcl-2 ratio to promote apoptosis; increases secretion of NO and TNF-α	Upregulation of NO and TNF-α secretion; Bax/Bcl-2 apoptotic pathway	Human breast cancer MCF-7 cells; RAW264.7 macrophages *in vitro*	*In vitro*	Intrinsic compound effect	Li et al. (2019)
Astragalus membranaceus Polysaccharide (AMP)	Inhibits solid tumor growth; improves body weight, spleen/thymus indices, and macrophage phagocytotic function; promotes serum secretion of IL-2, IL-12, and TNF-α, while decreasing IL-10 levels	Upregulation of IL-2/IL-12/TNF-α; Downregulation of IL-10	Murine H22 hepatocarcinoma model	*In vivo*	Intrinsic compound effect	Yang et al. (2013)
Astragalus Polysaccharide (APS)	Time-dependently binds to macrophages; increases production of NO and cytokines including TNF-α and GM-CSF; upregulates NF-κB protein levels	NF-κB signaling pathway	Murine RAW264.7 macrophages *in vitro*	*In vitro*	Intrinsic compound effect	Zhao et al. (2011)
Astragalus Polysaccharide (RAP)	Induces macrophage polarization toward the M1 phenotype; upregulates M1 markers (iNOS, IL-6, TNF-α, CXCL10); co-transplantation of RAP-induced BMDMs with tumor cells decreases tumor volume and weight	Notch signaling pathway	Mouse 4T1 breast cancer model; RAW264.7 macrophages; Mouse BMDMs	*In vivo*/*in* *vitro*	Intrinsic compound effect	Wei et al. (2019)
Astragalus Polysaccharide (APS)	Macrophage conditioned medium inhibits tumoroid growth and cell viability in a 3D tumor model; promotes cell apoptosis (nuclear fragmentation)	Upregulation of NO and TNF-α secretion	Human breast cancer 3D tissue-engineered tumor model (decellularized porcine lung scaffold); RAW264.7 macrophages	*In vitro* (3D culture model)	Intrinsic compound effect	Li et al. (2018)
Astragalus Polysaccharide (PG2)	Increases M1/M2 macrophage polarization ratio; downregulates IL-6 and IL-10 expression; time-dependently depletes tumor-associated M2 populations; synergistically enhances cisplatin efficacy and mitigates cachexia	M1/M2 polarization regulation; IL-6/IL-10 downregulation	NSCLC H441/H1299 cells *in vitro*; NSCLC patient clinical samples *ex vivo*; NSCLC mouse xenograft model *in vivo*	*In vitro*/*ex vivo*/*in vivo*	Intrinsic compound effect	Bamodu et al. (2019)
Cold-Water-Soluble Astragalus Polysaccharide (cAMPs-1A)	Oral administration significantly inhibits tumor growth; protects immune organs; promotes macrophage pinocytosis; improves percentages of lymphocyte subsets in peripheral blood	Macrophage pinocytosis and lymphocyte activation	Tumor-bearing mouse model	*In vivo*	Intrinsic compound effect	Liu et al. (2017)
Alcohol-soluble Astragalus Polysaccharide (ASP)	Significantly inhibits *in vivo* growth of H22 hepatoma cells; increases serum cytokines (TNF-α, IL-2, IFN-γ); enhances activities of macrophages, lymphocytes, and NK cells; induces tumor cell apoptosis	Elevation of serum cytokines (TNF-α, IL-2, IFN-γ); Immune cell activation	Mouse H22 hepatoma model	*In vivo*	Intrinsic compound effect	Yu et al. (2018)
Astragaloside IV (AS-IV)	Inhibits CT26 cell proliferation and induces apoptosis; reduces M2 and increases M1 macrophages; decreases TGF-β, IL-10, VEGF-A, and increases IFN-γ, IL-12, TNF-α; synergistic anti-tumor effects with aPD-1	M2 to M1 macrophage repolarization	Mouse CRC subcutaneous model (CT26); Bone marrow-derived macrophages *in vitro*	*In vivo*/*in* *vitro*	Intrinsic compound effect/Combination therapy effect	Liu et al. (2020)
Astragaloside IV (AS-IV) + anti-PD-1	Reduces tumor volume and weight; induces apoptosis and suppresses proliferation; increases M1 markers (mCD86), reduces M2 markers (mCD206), upregulates T cell activation markers (mCD69)	Inactivation of PI3K/Akt and ERK signaling pathways	Mouse Lewis lung carcinoma (LLC) subcutaneous model	*In vivo*	Combination therapy effect	Wu T. et al. (2024)
Astragaloside IV (AS-IV)	Reduces release of tumor-derived extracellular vesicles (TEVs) from CRC cells; remodels TAM polarization (decreases M2, increases M1); reduces liver metastasis and M2 macrophage infiltration *in vivo*	Downregulation of nSMase2 and Rab27a expression; Inhibition of M2 polarization	CRC cells (MC38); RAW264.7 macrophages; Mouse CRC spleen-to-liver metastasis model	*In vivo*/*in vitro*	Intrinsic compound effect	Zhou et al. (2024)

#### 3.1.2 Attenuation of immunosuppressive cellular networks

##### 3.1.2.1 Inhibition of regulatory T Cell accumulation and suppressive function

As a central immunosuppressive subset within the adaptive immune system, Tregs subvert their physiological role of maintaining systemic immune tolerance—a process typically mediated by key molecules like Cluster of Differentiation 25 (CD25), Cytotoxic T-Lymphocyte-Associated Protein 4 (CTLA-4), and Forkhead box P3 (Foxp3/FOXP3) (Liu et al., 2016; Tay et al., 2023)—to establish profound tumor-associated immunosuppression (Vignali et al., 2008; Tanaka and Sakaguchi, 2017). The TME actively recruits Tregs via chemokines like C-C Motif Chemokine Ligand 22 (CCL22) or TCR–MHC-II recognition, while tumor-induced metabolic alterations—characterized by high lactate and low glucose—selectively support Treg survival and function over effector CD4^+^ T cells (Liu et al., 2016). To attenuate anti-tumor immunity, Tregs utilize contact-dependent cytolysis and contact-independent suppression, which involves secreting inhibitory cytokines [TGF-β, IL-10, and Interleukin-35 (IL-35)], repressing IL-2 signaling, expressing inhibitory receptors like Lymphocyte Activation Gene 3 (LAG3), and utilizing the ectonucleotidase Cluster of Differentiation 39 (CD39) to convert extracellular ATP into suppressive adenosine (Liu et al., 2016; Wei et al., 2017; Tay et al., 2023). The pathways governing intra-tumoral Treg dominance closely mirror those maintaining systemic tolerance. Therefore, selectively targeting these localized suppressive mechanisms requires precise microenvironment-specific modulation. Future immunotherapies must achieve this delicate balance without inducing broad autoimmune toxicity.

In the context of tumor-induced immunosuppression, APS functions as an immunomodulator that attenuates Treg-mediated suppression. In in vitro studies utilizing cells derived from human HCC patients, APS has been shown to inhibit the proliferation of CD4^+^CD25^+^ Tregs. Furthermore, it interferes with the C-X-C Chemokine Receptor 4/C-X-C Motif Chemokine Ligand 12 (CXCR4/CXCL12) signaling pathway, which reduces Treg migration in these models and diminishes their immunosuppressive function (Li et al., 2012). Notably, while leveraging this APS-mediated interference presents valuable translational opportunities to prevent Treg infiltration and improve chemosensitivity, a clinical nuance remains. Successfully deploying such CXCR4/CXCL12 antagonists will require precision-targeted delivery strategies. It is critical to ensure that localized tumor remodeling occurs without inadvertently compromising broader systemic immune homeostasis (Kumagai et al., 2024).

Beyond oncology, APS demonstrates immunomodulatory effects in models of acute systemic inflammation. In a murine post-burn sepsis model, APS administration downregulates the key Treg transcription factor Foxp3 and the immunosuppressive factor IL-10 via Toll-Like Receptor 4 (TLR4) signaling. Consequently, it promotes a CD4^+^ T cell-mediated shift from a T Helper 2 (Th2) to a Th1 phenotype (Liu et al., 2011). Similarly, in murine models of polymicrobial sepsis, APS has been reported to reduce the Treg proportion and recalibrate both the Th17/Treg and Th1/Th2 balances (Hou et al., 2015). Intriguingly, although these acute models highlight the capacity of APS to modulate systemic immunosuppression, the dose-dependent nature of this intervention demands careful pharmacological optimization. Specifically, studies indicate that while moderate doses effectively promote immune balance, excessive APS administration can provoke hyperactive Th17 responses and subsequent organ injury. Therefore, extrapolating these systemic findings into chronic tumor microenvironments warrants rigorous dose titration and context-specific validation.

Addressing metabolic immunosuppression, AS-IV has demonstrated the capacity to modulate Indoleamine 2,3-Dioxygenase (IDO)—a key enzyme in tryptophan catabolism and a major driver of tumor-associated immune escape. In an orthotopic murine model utilizing IDO-overexpressing Lewis lung carcinoma cells, AS-IV administration was shown to significantly suppress tumor growth (Zhang et al., 2014). This therapeutic effect is associated with a concurrent modulation of the T-cell compartment: it decreases the frequency of immunosuppressive Tregs while simultaneously enhancing CTL activity both *in vitro* and *in vivo*. Alongside these cellular shifts, AS-IV treatment blocks IDO induction, suggesting its potential to attenuate the IDO–Treg axis and remodel the TIME toward a more immunostimulatory state (Zhang et al., 2014). Intriguingly, while AS-IV successfully mitigates IDO expression in these specific murine models, a critical pharmacological nuance requires further clarification. It remains mechanistically ambiguous whether AS-IV acts as a direct competitive inhibitor structurally binding to the IDO enzyme, or if this observed IDO downregulation merely represents a secondary consequence of disrupted upstream inflammatory signals. Clarifying this precise molecular engagement will reliably inform future targeted combinations.

Although both compounds effectively mitigate Treg-mediated immunosuppression in preclinical models, they rely on distinct pharmacological trajectories; APS rebalances systemic immunity and chemokine signaling, in contrast to AS-IV, which interferes with metabolic checkpoints like IDO. By reducing the accumulation and suppressive function of these regulatory cells in experimental models, these immunomodulators demonstrate the potential to restore cytotoxic T cell activity and counteract TME-induced immune tolerance. Advancing these interventions into clinical practice, however, requires rigorous dose optimization, precision-targeted delivery, and the validation of direct molecular targets within physiologically authentic models (Table 5).

**TABLE 5:** Experimental evidence-based summary of *Astragalus* extracts on suppressing regulatory T Cells (Tregs) in tumors and inflammatory diseases.

Compound/formulation	Immunological effects	Molecular targets/pathways	Tumor models/systems	Level of evidence	Effect attribution	References
Astragalus Polysaccharides (APS)	Inhibits the proliferation and migration of CD4^+^CD25^+^ Treg cells; reduces FOXp3 mRNA expression; restores the cytokine balance in the tumor microenvironment; blocks Treg cell chemotaxis to the tumor site	CXCR4/CXCL12 signaling pathway (SDF-1/CXCR4 axis)	Fresh tissue samples from 31 HCC patients; *In vitro* Treg cell proliferation and migration assays	*Ex vivo*/*in vitro*	Intrinsic compound effect	Li et al. (2012)
Astragalus Polysaccharides (APS)	Decreases Foxp3 expression and IL-10 production in CD4^+^CD25^+^ Tregs of burned septic mice; restores CD4^+^ T cell proliferative activity and IL-2/IL-2Rα expression; triggers a Th2 to Th1 shift; effects are blocked by anti-TLR4 antibodies	TLR4 signaling pathway	Mouse postburn *P. aeruginosa* infection sepsis model	*In vivo*	Intrinsic compound effect	Liu et al. (2011)
Astragalus Polysaccharides (APS)	Downregulates the percentages of circulating Th2 cells and Tregs in septic mice; restores Th cell populations and the percentage of activated Th cells; high-dose (>200 mg/kg) upregulates Th17 cells, leading to excessive inflammation and organ injury	Th1/Th2/Th17/Treg balance regulation	Mouse cecal ligation and puncture (CLP) polymicrobial sepsis model	*In vivo*	Intrinsic compound effect (Dose-dependent bidirectional regulation)	Hou et al. (2015)
Astragaloside IV (AS-IV)	Downregulates regulatory T cells (Tregs) frequency; upregulates cytotoxic T lymphocytes (CTLs) activity; blocks IDO induction both *in vitro* and *in vivo*	IDO expression inhibition	IDO-overexpressed murine Lewis lung carcinoma orthotopic model	*In vivo*/*in vitro*	Intrinsic compound effect	Zhang et al. (2014)

##### 3.1.2.2 Suppression of MDSC expansion and recruitment

Within the TME, MDSCs emerge as a highly heterogeneous population of immature myeloid cells, broadly categorized into polymorphonuclear (PMN-MDSC) and monocytic (M-MDSC) subtypes (Gabrilovich, 2017). These cells expand rapidly under tumor-driven conditions to establish an immunosuppressive milieu that promotes metastasis and limits the efficacy of immunotherapy (Gabrilovich, 2017; Li M. O. et al., 2021). Functioning primarily to broadly inhibit T-cell responses, PMN-MDSCs mediate suppression utilizing arginase, ROS, and prostaglandin E2 (PGE2) (Rodriguez et al., 2005; Donkor et al., 2009; Mao et al., 2013)—a capacity further enhanced when the uptake of senescent tumor-released mitochondrial DNA activates their cGAS–STING–NF-κB pathway (Lai et al., 2025). Concurrently, M-MDSCs suppress T-cell function primarily via NO production (Veglia et al., 2018) and possess the ability to rapidly differentiate into immunosuppressive M2-type macrophages (Kwak et al., 2020). The pathological expansion and mobilization of these subsets are tightly regulated by signaling networks involving Interferon Regulatory Factor 8 (IRF8), CCAAT/Enhancer Binding Protein Beta (C/EBPβ), and critically, Signal Transducer and Activator of Transcription 3 (STAT3) (Wu et al., 2022). By upregulating anti-apoptotic and proliferative genes—such as B-Cell Lymphoma-extra Large (Bcl-Xl), Cellular Myc Proto-Oncogene (c-Myc), and cyclin D—STAT3 arrests normal myeloid differentiation (Lee et al., 2013; Yu et al., 2014), and can further drive IL-6-dependent PMN-MDSCs to secrete exosomal miR-93-5p, which polarizes M-MDSCs toward the M2 phenotype (Wang Y. et al., 2023). Because these pathological suppressors share significant morphological features with normal myeloid progenitors, selectively targeting this STAT3-driven network without disrupting healthy systemic myelopoiesis represents an essential objective for precision pharmacology.

APS exhibits context-dependent immunomodulatory properties, regulating MDSC activity through a highly adaptable “signal intervention–metabolic remodeling” cascade. In murine lung cancer metastasis models, APS has been shown to modulate the pre-metastatic niche by inhibiting the Sphingosine-1-Phosphate Receptor 1 (S1PR1)–STAT3 axis. This targeted blockade effectively curtails MDSC recruitment and accumulation to deliver potent antitumor effects (Shen M. et al., 2023). Conversely, in murine models of EAE, APS exerts a protective effect by promoting MDSC suppressive functions to attenuate pathological inflammation. It activates the AMP-Activated Protein Kinase (AMPK)/Janus Kinase (JAK)/STAT3 pathway to upregulate arginase-1 (Arg-1) expression in MDSCs. This metabolic modulation enhances their capacity to inhibit overactive CD4^+^ T cells, ultimately alleviating neuroinflammation (Wang et al., 2025). While this dual capacity to either suppress or empower MDSCs via STAT3-related pathways highlights the context-dependent profile of APS (Shen M. et al., 2023; Wang et al., 2025), the precise molecular switches governing this behavior require further experimental elucidation.

The pharmacological reach of APS extends to the gut-immune axis, establishing an indirect mechanism to restrain MDSC accumulation. In murine melanoma models, APS administration significantly reduces MDSC accumulation in tumor tissues and downregulates the expression of associated suppressive molecules, including Arg-1, IL-10, and TGF-β (Ding et al., 2021). Consequently, this reduction in immunosuppression promotes the tumor-killing function of CD8^+^ T cells. Mechanistically, these effects are mediated by the modulation of the gut microbiota. Specifically, APS enriches beneficial microbes such as Bifidobacterium pseudolongum and *Lactobacillus* johnsonii. This microbial shift consequently elevates circulating metabolites like glutamate and creatine to exert systemic tumor control (Ding et al., 2021).

Ultimately, although modulating MDSC populations and their associated signaling networks presents a promising therapeutic avenue, the translation of these preclinical findings into clinical applications must account for the extreme phenotypic heterogeneity and disease-specific roles of MDSCs across different pathological states (Table 6).

**TABLE 6:** Experimental evidence-based summary of *Astragalus* polysaccharides on inhibiting myeloid-derived suppressor cells (MDSCs) in tumors and metastasis.

Compound/formulation	Immunological effects	Molecular targets/pathways	Tumor models/systems	Level of evidence	Effect attribution	References
Astragalus Polysaccharide (APS)	Inhibits the formation of the premetastatic niche (PMN) in the lung; suppresses the recruitment and accumulation of MDSCs in the lung	S1PR1-STAT3 signaling pathway (downregulation of S1PR1, STAT3, and p-STAT3 expression)	Mouse tumor lung metastasis model	*In vivo*	Intrinsic compound effect	Shen M. et al. (2023)
Astragalus Polysaccharide (APS)	Reduces the accumulation of MDSCs and the expression of Arg-1, IL-10, and TGF-β in tumor tissues; enhances the tumor-killing function of CD8^+^ T cells; the MDSC-reducing effect is abolished upon co-administration of antibiotics	Remodeling of gut microbiota (enrichment of *Bifidobacterium pseudolongum*, *Lactobacillus johnsonii*, etc.); Alteration of fecal metabolites (elevation of glutamate and creatine)	Mouse melanoma model; Antibiotic treatment validation; Fecal microbiota transplantation	*In vivo*	Intrinsic compound effect (indirect regulation of MDSCs via the gut-immune axis)	Ding et al. (2021)

##### 3.1.2.3 Reprogramming of pro-tumorigenic M2 macrophages

Within the TME, TAMs are frequently skewed toward an alternatively activated M2 state (Asai et al., 2017) by specific signaling molecules—primarily Macrophage Colony-Stimulating Factor (M-CSF), IL-4, IL-10, IL-13, and TGF-β (Anderson et al., 2021). Depending on the context, this M2 population segregates into functionally distinct subtypes (Wang L. et al., 2021) that actively drive disease progression at primary and metastatic sites by promoting basement-membrane remodeling, stimulating angiogenesis, and inducing broad immunosuppression (Quail and Joyce, 2013; Caux et al., 2016), largely through the secretion of pro-tumor factors like IL-10, TGF-β, and C-C Motif Chemokine Ligand 17 (CCL17) (Biswas et al., 2013). Clinically, elevated M2 levels or a decreased lymphocyte-to-monocyte ratio (LMR) correlate significantly with poor patient outcomes (Yuan et al., 2014; Gu et al., 2016), prompting contemporary therapeutic strategies for malignancies like CRC and ovarian cancer to prioritize reprogramming TAMs back toward an anti-tumorigenic M1 phenotype (Wang X. et al., 2021; Zhou et al., 2024). However, because macrophage phenotypes exist on a continuous spectrum rather than in rigid binary states, safely navigating this dynamic plasticity to stabilize M1 activation without triggering systemic inflammatory toxicity represents a pivotal pharmacological objective.

Astragalus Polysaccharide (APS) effectively counteracts the M2-driven immunosuppressive milieu characteristic of the TME. In murine models of NSCLC, the administration of clinical-grade APS (PG2) actively decreased the proportion of M2 macrophages by reducing tumor-derived IL-6 and IL-10 expression. This intervention effectively depletes the tumor-associated M2 population and has been shown to enhance the chemotherapeutic efficacy of cisplatin (Bamodu et al., 2019). Similarly, in preclinical models of bladder cancer, APS attenuated cisplatin (DDP) resistance by inhibiting the PI3K/AKT/Forkhead Box O1 (FoxO1) axis. While this targeted blockade induces tumor cell apoptosis, bioinformatic analyses further suggest it may concurrently modulate CD8^+^ T cell exhaustion and M2 polarization (Chen et al., 2025). While APS exhibits synergistic potential with cisplatin to improve anti-tumor immunity in these models (Bamodu et al., 2019; Chen et al., 2025), the exact sequence of these events requires careful pharmacological dissection. It remains mechanistically ambiguous whether this observed M2 depletion results from APS directly binding to macrophage receptors, or if it merely represents a secondary consequence of PI3K-driven tumor cell apoptosis and reduced inflammatory signaling. Furthermore, at the epigenetic level, APS has been reported to exhibit anti-proliferative effects in ovarian cancer cells. *In vitro* studies indicate that it downregulates oncogenic microRNAs to restore the miR-27a/F-Box and WD Repeat Domain Containing 7 (miR-27a/FBXW7) axis, thereby mediating tumor-suppressive activity (Guo Y. et al., 2020).

The elemental composition of APS—independent of its direct effects on intracellular signaling—acts as a key contributing factor to its immunomodulatory profile. In murine H22 ascites hepatoma and S180 sarcoma models, Se-PAM efficiently inhibit the M2-like polarization of TAMs and enhance CD8^+^ T cell cytotoxicity. Notably, because this robust immunomodulation is highly selenium-dependent (Li et al., 2014), the natural geographical variation of trace elements in Astragalus roots introduces a distinct translational variable. Achieving consistent clinical outcomes will likely require standardized trace-element fortification or quantification during APS extraction. Moreover, preclinical strategies are now exploiting APS as an active structural matrix for advanced drug delivery. In murine ectopic and metastatic models of ovarian cancer, methotrexate-modified, podophyllotoxin-loaded APS micelles (MTX-PPT-micelles) actively target folate receptors. This nano-platform not only delivers cytotoxic payloads but also promotes the repolarization of TAMs from an M2 to an M1 phenotype (Liu et al., 2025b). Crucially, although this dual-action platform successfully alleviates local immunosuppression in these studies, rigorously decoupling the intrinsic M1-polarizing effects of the APS vector from the pharmacological cytotoxicity of podophyllotoxin remains an essential objective for rationally optimizing these advanced nano-formulations.

Across various preclinical cancer models, AS-IV exerts a two-pronged regulatory effect on M2-driven immunosuppression, encompassing both direct macrophage reprogramming and indirect microenvironmental modulation. Regarding direct cellular interactions, AS-IV has been shown to attenuate M2 polarization in models of LC and cervical cancer. Specifically, in in vitro and murine Lewis lung cancer models, it inhibits IL-4/IL-13–induced M2 polarization and downregulates Cluster of Differentiation 206 (CD206) by suppressing AMPKα activation (Xu et al., 2018). Similarly, in in vitro models of cervical cancer, AS-IV treatment reduces the expression of key M2 markers, including Cluster of Differentiation 163 (CD163), CD206, IL-10, and TGF-β. Conditioned medium from these treated macrophages (AS-IV-M2-CM) exhibits a diminished capacity to drive epithelial-mesenchymal transition (EMT) and angiogenesis in cervical cancer cells. This inhibitory effect is mediated by the inactivation of the TGF-β/Mothers Against Decapentaplegic Homolog 2/3 (Smad2/3) signaling pathway (Shen L. et al., 2023). In contrast to its direct action on macrophages, AS-IV also exerts indirect immunomodulatory effects by targeting CRC cells. In murine metastasis models, it downregulates nSMase2 and Rab27a expression in tumor cells to reduce the release of tumor-derived extracellular vesicles (TEVs). By limiting this vesicular transport, AS-IV decreases the delivery of pro-M2 signals to TAMs, thereby promoting a restorative shift toward an M1 phenotype *in vivo* (Zhou et al., 2024).

Although these *in vitro* and *in vivo* models illustrate the capacity of AS-IV to disrupt macrophage-tumor cross-talk (Xu et al., 2018; Shen L. et al., 2023), the living tumor microenvironment involves a highly complex signaling network. Because AS-IV acts as an AMPK inhibitor in macrophages while functioning as an indirect TEV suppressor in tumor cells, clarifying its primary pharmacological target in human physiology remains an essential task for optimizing future clinical applications. Overall, current data suggest that the diverse pharmacological actions of AS-IV converge on the attenuation of M2 polarization, which effectively restricts the accumulation of pro-tumorigenic factors like TGF-β, IL-10, and VEGF.

In summary, current preclinical models demonstrate that both APS and AS-IV systematically counteract TAM-mediated immunosuppression, albeit through distinct pharmacological profiles. Whereas AS-IV primarily disrupts precise intercellular signaling and vesicular cross-talk, the immunomodulatory efficacy of APS is linked to its structural matrix and elemental composition. Despite these divergent operational pathways, both agents functionally converge on repolarizing the macrophage compartment to reverse TME-induced immune tolerance. However, translating these multifaceted interventions into clinical practice involves significant pharmacological complexities. Resolving the ambiguity between primary molecular targets and secondary microenvironmental adaptations, alongside managing compositional variables such as trace-element dependency, remain critical hurdles. To advance these therapeutic strategies toward clinical application, future investigations should prioritize rigorous target decoupling within physiologically authentic models and establish standardized extraction protocols to reliably evaluate their therapeutic consistency (Table 7).

**TABLE 7:** Experimental evidence-based summary of *Astragalus* extracts on repolarizing or inhibiting M2 macrophages in tumors.

Compound/formulation	Immunological effects	Molecular targets/pathways	Tumor models/systems	Level of evidence	Effect attribution	References
Astragalus Polysaccharide (APS)	Inhibits BLCA cell proliferation and induces apoptosis; induces G0/G1 cell cycle arrest; enhances DDP-induced apoptosis; modulates M2 polarization and CD8^+^ T cell exhaustion to overcome DDP resistance	PI3K/AKT/FoxO1 axis (downregulation of PI3K-p110β and p-AKT, upregulation of FoxO1); Downregulation of CCND1	Bladder cancer cell lines *in vitro*; Mouse tumor model *in vivo*	*In vivo*/*in vitro*	Intrinsic compound effect	Chen et al. (2025)
HP-NPs@PTX/Bai (APS-S-Pae + oHA-Man-FA)	GSH/ROS dual-responsive TAMs-targeted nanoparticles; re-educates TAMs from M2 to M1 phenotype; synergistically co-delivers PTX and baicalin to enhance anti-tumor efficacy and ameliorate PTX toxicity	TAMs re-education (M2 to M1 phenotype shift)	Mouse breast cancer model (targeted delivery system)	*In vivo*/*in vitro*	Nanoplatform + Combination therapy effect (APS acts as a structural matrix; Baicalin possesses TAM-regulating activity)	Guo C. et al. (2022)
Selenium-containing Astragalus Polysaccharides (Se-PAMs)	Selenium-dependently suppresses M2-like polarization of TAMs; enhances NK and CD8^+^ T cell cytotoxic activities; improves peritoneal macrophage phagocytosis; ameliorates CD4^+^ T cell apoptosis	Selenium-dependent immunomodulation	Mouse H22 ascitic hepatoma and S180 sarcoma models	*In vivo*	Intrinsic compound effect (immunomodulatory activity is highly dependent on selenium content)	Li et al. (2014)
MTX-PPT-micelles (APS-PDA vector)	MTX-modified APS micelles target ovarian cancer cells with high folate receptor expression; shifts TAMs from M2 to M1 phenotype; inhibits tumor proliferation, invasion, and metastasis; improves the tumor immunosuppressive microenvironment	TAMs repolarization (M2 to M1)	Mouse ovarian cancer ectopic and lung metastasis models	*In vivo*/*in vitro*	Nanoplatform effect (APS as the structural matrix, co-loaded with PPT, MTX-targeted modification)	Liu et al. (2025b)
Astragaloside IV (AS-IV)	Inhibits IL-4/IL-13-induced M2 macrophage polarization (decreases CD206 and M2-associated gene expression); suppresses M2-CM-induced lung cancer cell invasion, migration, and angiogenesis; reduces M2 macrophage percentage and the number of Lewis lung cancer metastases *in vivo*	AMPKα signaling pathway inhibition	Human THP-1 monocyte-derived M2 macrophages; A549/H1299 lung cancer cells; Mouse Lewis lung cancer model	*In vivo*/*in vitro*	Intrinsic compound effect	Xu et al. (2018)
Astragaloside IV (AS-IV) + anti-PD-1	Reduces M2 macrophages and increases M1 macrophages; decreases TGF-β, IL-10, and VEGF-A; increases IFN-γ, IL-12, and TNF-α; combination with aPD-1 enhances T cell infiltration	M2 to M1 macrophage repolarization	Mouse CRC subcutaneous model (CT26)	*In vivo*/*in vitro*	Combination therapy effect (AS-IV + anti-PD-1)	Liu et al. (2020)
Astragaloside IV (AS-IV)	Reduces tumor-derived extracellular vesicle (TEV) release; conditioned medium reshapes TAM polarization (decreases M2, increases M1), leading to reduced cancer cell invasion and migration; reduces M2 macrophage infiltration in liver metastases	nSMase2/Rab27a pathway (downregulation)	CRC cells (MC38); RAW264.7 macrophages; Mouse CRC spleen-to-liver metastasis model	*In vivo*/*in vitro*	Intrinsic compound effect	Zhou et al. (2024)
Astragaloside IV (AS-IV)	Inhibits M2 polarization (decreases CD163, IL-10, TGF-β, and CD206); CM from AS-IV-treated M2 macrophages notably inhibits angiogenesis, migration, and EMT in cervical cancer cells	Inactivation of TGF-β/Smad2/3 signaling	M2 macrophages; Cervical cancer (CC) cells	*In vitro*	Intrinsic compound effect	Shen L. et al. (2023)
Astragaloside IV (AS-IV)	Decreases the expression of M2 macrophage markers; inhibits the pro-tumor effect (proliferation, migration, invasion) of M2 macrophages; significantly reduces tumor volume and weight *in vivo*	TLR4/NF-κB/STAT3 signaling pathway (downregulation)	Human THP-1-derived M2 macrophages; HCC cells (Huh-7); Mouse subcutaneous tumor model	*In vivo*/*in vitro*	Intrinsic compound effect	Min et al. (2022)
Astragaloside IV (AS-IV)	Suppresses macrophage polarization to M2 phenotype; inhibits M2 macrophage-induced breast cancer cell progression (proliferation, migration, and invasion)	Deactivation of the Akt/Foxo1 signaling pathway via suppressing TGF-β	Macrophages; Breast cancer (BC) cells	*In vitro*	Intrinsic compound effect	Yu et al. (2023)
Astragaloside IV (AS-IV)	Suppresses IL-4/IL-13-induced macrophage M2 polarization (decreases CD14^+^CD206^+^ ratio and M2 markers); antagonizes M2 macrophage co-culture-evoked cell proliferation, invasion, and migration	HMGB1-TLR4 signaling (suppression)	THP-1 macrophages; Ovarian cancer cells	*In vitro*	Intrinsic compound effect	Wang X. et al. (2021)

##### 3.1.2.4 Modulation of auxiliary type-2 immunosuppressive networks

**FIGURE 3:**
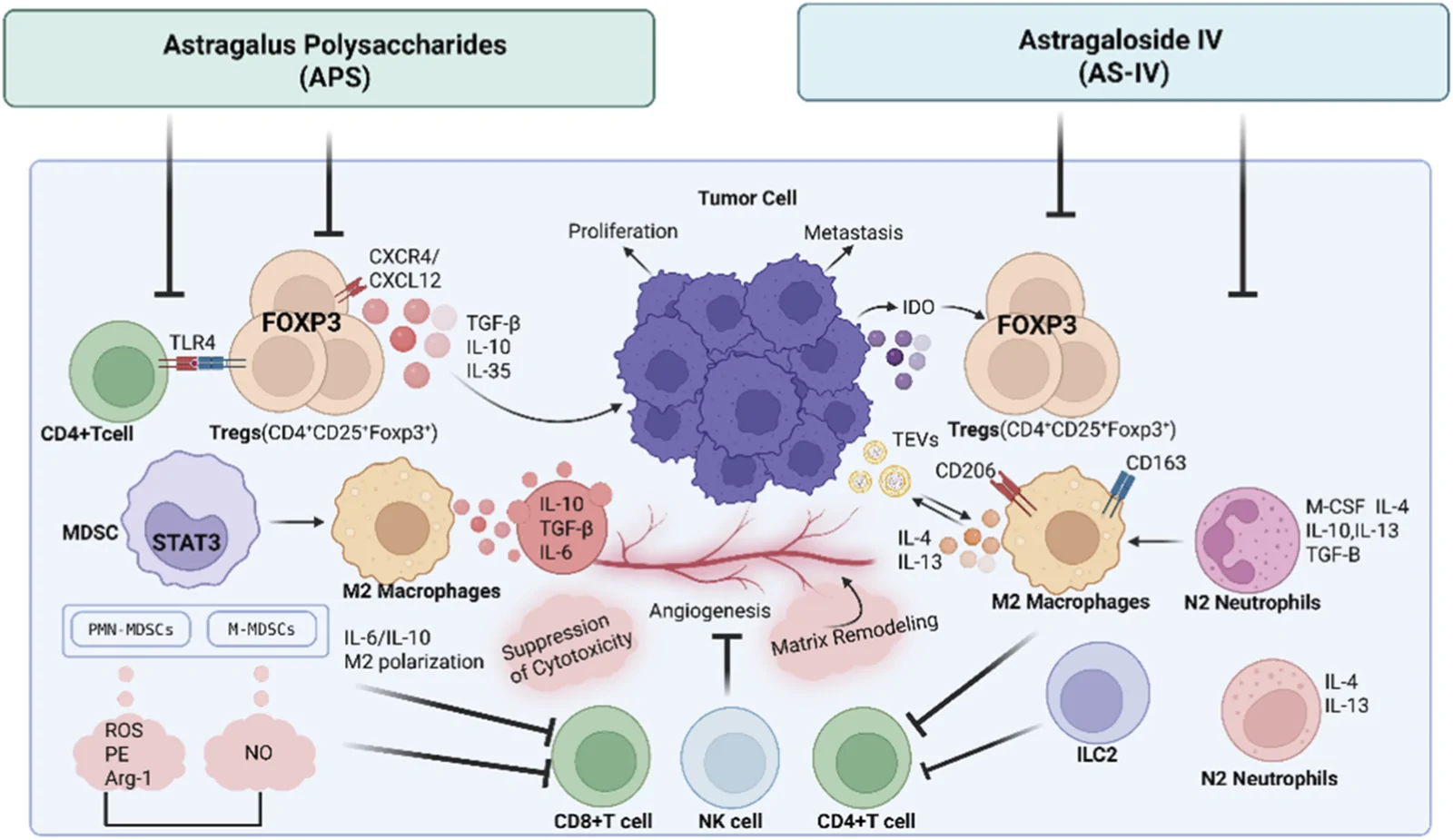
Modulation of the immunosuppressive network within the TIME by APS and AS-IV in preclinical models. This schematic delineates the cellular networks that attenuate anti-tumor immune responses and facilitate tumor proliferation and metastasis. Driven by STAT3 signaling, MDSC subsets (PMN-MDSCs and M-MDSCs) utilize ROS, NO, and Arg-1 to suppress the activity of cytotoxic effectors, including CD8^+^ T cells, CD4^+^ T cells, and NK cells. Concurrently, FOXP3-expressing Tregs are recruited to the tumor bed via the CXCR4/CXCL12 axis. By interacting with tumor-associated IDO and secreting inhibitory cytokines (TGF-β, IL-10, IL-35), these Tregs further promote immune tolerance. Additionally, alternatively activated M2 macrophages (CD206^+^/CD163^+^), induced by IL-6/IL-10, IL-4/IL-13, and tumor-derived TEVs, secrete factors that mediate angiogenesis and matrix remodeling. Parallel contributions from N2 neutrophils and ILC2s supply type 2 cytokines (IL-4, IL-13) to sustain this suppressive milieu. Based on current experimental evidence, APS (green box) attenuates these suppressive networks by inhibiting STAT3 signaling in MDSCs, modulating TLR4 signaling to reduce FOXP3 expression and CXCR4/CXCL12-mediated Treg recruitment, and restricting IL-6/IL-10-driven M2 polarization. In parallel, AS-IV (cyan box) interferes with the IDO–Treg axis and mitigates M2 polarization driven by IL-4/IL-13, TEVs, and N2 neutrophil-derived factors. Collectively, these modulatory effects highlight the potential of APS and AS-IV to counteract microenvironmental immunosuppression, thereby restoring cytotoxic effector function and restricting tumor progression in experimental settings.

In addition to the primary macrophage and T-cell axes, the TIME is characterized by the accumulation of type-2 skewed effector populations, including N2-polarized neutrophils, NKT2 cells, and ILC2s (Figure 3).

N2 neutrophils, which are predominantly driven toward a pro-tumorigenic state by TGF-β (Fridlender et al., 2009), contribute to immunosuppression and tumor progression through several distinct mechanisms. These cells can impair anti-tumor immune responses by releasing ROS and reactive nitrogen species (RNS) (Güngör et al., 2010), while simultaneously inducing CD8^+^ T-cell apoptosis via TNF-α and NO-dependent pathways (Michaeli et al., 2017). Structurally, they facilitate extracellular matrix remodeling through the secretion of matrix metalloproteinases (MMPs) (Ardi et al., 2007), which stabilizes integrins and promotes cancer-cell migration and invasion (Spiegel et al., 2016; Das et al., 2017). Additionally, N2 neutrophils coordinate with other immunosuppressive populations by recruiting M2 macrophages and Tregs into the TME (Giese et al., 2019).

Type 2 natural killer T (NKT2) cells also represent a significant component of this type-2 microenvironment (Hinshaw and Shevde, 2019). Under the regulation of the transcription factor GATA Binding Protein 3 (Gata3), NKT2 cells secrete IL-4 and IL-13, which are associated with pro-tumorigenic outcomes (Yasuoka et al., 2016). Accordingly, experimental and emerging clinical observations suggest that selectively depleting this specific NKT2 subpopulation correlates with tumor regression, highlighting their role in immune evasion (Dhodapkar and Kumar, 2017).

Group 2 innate lymphoid cells (ILC2s) further contribute to the shaping of the type-2 microenvironment (Bie et al., 2014; Trabanelli et al., 2019). Their immunomodulatory activity involves the secretion of Interleukin-5 (IL-5), IL-4, and IL-13 (Fu et al., 2021), which facilitates the recruitment and activation of MDSCs (Jovanovic et al., 2014; Chevalier et al., 2017; Trabanelli et al., 2017). This establishes a local feedback loop, as the activated MDSCs subsequently produce TGF-β to drive further M2 macrophage polarization (Gong et al., 2012). ILC2s also produce amphiregulin, which has been reported to support Treg expansion (Zaiss et al., 2013; Zaiss et al., 2015). Cumulatively, the cytokine networks established by these type-2 effectors can significantly attenuate NK cell-mediated immune surveillance, further promoting a permissive environment for tumor growth (Long et al., 2018).

While the preclinical modulation of macrophages and Tregs by APS and AS-IV has been increasingly well-characterized, their direct pharmacological impact on these N2, NKT2, and ILC2 populations remains largely unexplored in the current literature. This knowledge gap highlights a relevant avenue for future investigation. Given that the survival and functional cross-talk of these type-2 effectors depend significantly on IL-4, IL-13, and TGF-β—cytokines that APS and AS-IV have been shown to attenuate in various experimental models—it is mechanistically plausible that these compounds exert indirect regulatory effects on this network. Consequently, determining whether APS and AS-IV possess the capacity to directly modulate these emerging cellular targets, or if their influence is primarily a secondary consequence of cytokine reduction, represents an important objective for clarifying their broader immunomodulatory profiles.

### 3.2 Disruption of metabolic competition and bioenergetic reprogramming

The TME represents a metabolically restrictive niche characterized by acidosis, hypoxia, and metabolite accumulation (Bader et al., 2020). Within this ecosystem, tumor cells frequently exhibit the Warburg effect, preferentially utilizing aerobic glycolysis (Warburg et al., 1927; Eagle, 1955; Warburg, 1956; Márquez et al., 1989; Pavlova and Thompson, 2016) to outcompete anti-tumor immune cells for essential resources like glucose and glutamine (DeBerardinis and Chandel, 2016; Pavlova and Thompson, 2016). This profound nutrient depletion establishes a functional dichotomy based on distinct cellular metabolic preferences: while activated T cells, TLR-stimulated DCs, and M1-like macrophages primarily depend on glycolysis, *de novo* lipogenesis, and amino-acid metabolism to support their effector functions (Pearce et al., 2013; Pearce and Everts, 2015; Netea-Maier et al., 2018; Bader et al., 2020), immunosuppressive populations such as Tregs, M2-like macrophages, and MDSCs preferentially engage oxidative phosphorylation (OXPHOS) and fatty-acid oxidation (FAO) (Michalek et al., 2011; Beier et al., 2015; Hossain et al., 2015; Ho and Liu, 2016; Netea-Maier et al., 2018). Consequently, the glucose-deprived TME structurally drives T-cell exhaustion while providing a selective bioenergetic advantage for regulatory subsets (Bader et al., 2020). Because the metabolic plasticity of immune cells often allows them to adapt via alternative compensatory pathways, rationally overcoming this immunosuppression generally requires shifting from absolute metabolic blockades toward precise metabolic modulation. Selectively enhancing the bioenergetic fitness of anti-tumor effectors within this restrictive milieu represents a critical therapeutic objective.

Current experimental evidence indicates that APS and AS-IV modulate tumor bioenergetics by interfering with key pathways in lipid and glucose metabolism. In both *in vitro* and *in vivo* models of prostate cancer, APS demonstrates an ability to disrupt lipid metabolism. It upregulates miR-138-5p to repress Sirtuin 1 (SIRT1) and activate AMPK signaling. This cascade inhibits the nuclear translocation of Sterol Regulatory Element-Binding Protein 1 (SREBP1), thereby downregulating lipogenic transcriptional programs and depleting intracellular triglyceride and cholesterol pools (Guo S. et al., 2020). While this non-coding RNA-mediated metabolic modulation limits proliferation and invasion in these specific prostate models, a distinct mechanistical question arises. It remains to be determined whether APS interacts directly with the miR-138-5p processing machinery or if this epigenetic shift represents a secondary systemic response to altered cellular stress.

Similarly, AS-IV influences glucose metabolism through both transcriptional and post-translational regulation. In a rat model of gastric precancerous lesions, AS-IV upregulates miR-34a to suppress a suite of glycolytic drivers, including Lactate Dehydrogenase A (LDHA), Monocarboxylate Transporter 1/4 (MCT1/4), and HIF-1α (Zhang et al., 2018). In cell lines and xenograft models of HCC, AS-IV modulates metabolic function by targeting protein succinylation. It downregulates the succinyltransferase Lysine Acetyltransferase 2A (KAT2A), which reduces phosphoglycerate mutase 1 (PGAM1) K161 succinylation. This specific post-translational modification correlates with decreased glycolytic flux and impairs cell viability (Zhu and Lu, 2024).

Although these diverse interventions consistently converge on a common cascade—targeting metabolic enzymes via microRNAs or post-translational modifications—their translational optimization requires caution. Because enzymes like LDHA and PGAM1 are also essential for healthy regenerative tissues, future research should evaluate whether APS and AS-IV can selectively modulate these metabolic nodes within the tumor without compromising systemic energy homeostasis.

## 4 Discussion

### 4.1 Immunometabolic modulation by APS and AS-IV in the tumor microenvironment

Current preclinical evidence demonstrates that APS and AS-IV modulate the TME not as broad-spectrum stimulants, but through targeted interventions across distinct immune and metabolic networks. By integrating the direct priming of immune effectors with the deconstruction of pathological metabolic crosstalk, these natural constituents offer a sophisticated, multi-node pharmacological framework for restoring microenvironmental homeostasis. Within the cellular compartment, the regulatory effects of APS and AS-IV are characterized by their specific cellular targets and downstream signaling cascades observed in distinct experimental settings.

Specifically, APS primarily serves to reinvigorate immune surveillance by driving the maturation of APCs and preventing lymphocyte exhaustion. In murine models of HCC, APS facilitates the adaptive immune axis by supporting the persistence of engineered Glypican-3 (GPC3)-targeted CAR-T cells, promoting the formation of the CD122^+^CXCR3^+^ memory phenotype and attenuating exhaustion via the downregulation of PD-1 (Zhang et al., 2024). In the innate immune compartment, APS drives macrophage polarization toward a tumoricidal M1 state via the Notch signaling pathway in murine breast cancer models (Wei et al., 2019). Furthermore, APS acts as a potent immunological adjuvant—both as an unmodified compound acting via NO signaling (Zhu N. et al., 2016) and within engineered nanoplatforms (Xiong et al., 2020)—to facilitate DC maturation by upregulating MHC-II and costimulatory molecules (CD80/86).

Conversely, AS-IV primarily serves to intercept pro-tumorigenic communication networks, alleviating myeloid-derived suppression and facilitating downstream cytotoxic responses. In CRC metastasis models, AS-IV disrupts tumor-macrophage crosstalk by downregulating the nSMase2/Rab27a axis, thereby inhibiting the release of TEVs that drive M2-like polarization (Zhou et al., 2024). Concurrently, in *ex vivo* healthy donor models, AS-IV upregulates DC costimulatory markers to enhance specific CTL responses against HCC cells (Guo H. et al., 2022). While AS-IV is also associated with enhanced CTL infiltration in TNBC models when utilized in multi-agent nano-delivery systems, it is crucial to clarify that the activation of associated pathways, such as cGAS-STING, is frequently driven by carrier-specific bioactivity (e.g., the co-release of manganese ions) rather than the intrinsic direct agonism of the saponin alone (Wang et al., 2024).

Beyond direct immune activation, APS and AS-IV interfere with specific metabolic checkpoints, although these interventions appear highly dependent on the tumor’s baseline bioenergetic profile. For example, in prostate cancer models, APS selectively disrupts lipid metabolism by modulating the miR-138-5p/SIRT1/SREBP1 pathway, leading to the depletion of intracellular triglyceride and cholesterol pools (Guo S. et al., 2020). Conversely, AS-IV targets aerobic glycolysis—the Warburg effect—in gastric precancerous lesions and HCC models by upregulating miR-34a to suppress LDHA (Zhang et al., 2018) and by modulating KAT2A-mediated PGAM1 succinylation (Zhu and Lu, 2024), respectively, thereby restricting glycolytic flux and lactate production.

While these metabolic shifts potentially alleviate the resource competition that impairs immune effectors (Pearce et al., 2013; Pearce and Everts, 2015; Netea-Maier et al., 2018; Bader et al., 2020), the precise hierarchy of these events remains to be fully elucidated. Specifically, it is currently inferential whether the suppression of lactate by AS-IV acts as the primary driver of immune restoration or functions as a permissive microenvironmental condition for subsequent immune activation. Furthermore, given that immunosuppressive populations such as Tregs and M2 macrophages rely heavily on FAO (Michalek et al., 2011; Beier et al., 2015; Hossain et al., 2015; Ho and Liu, 2016; Netea-Maier et al., 2018), it is mechanistically plausible that the lipid-modulating effects of APS may selectively restrict their accumulation; however, direct experimental confirmation of this causal link within a unified solid tumor model is still required.

Ultimately, the potential synergy between APS and AS-IV—integrating APS-mediated immune priming with AS-IV-mediated de-repression of suppressive barriers—offers a rational framework for precision immunotherapy. However, translating these preclinical observations into clinical practice requires significant caution. Because much of the current evidence is derived from rodent-based xenografts or *ex vivo* models, which may not fully recapitulate the dense stroma and severe hypoxia of human solid tumors, future research must prioritize the validation of these molecular targets in high-fidelity patient-derived models.

### 4.2 Spatiotemporal dynamics and high-resolution profiling of the APS/AS-IV regulatory network

The TIME functions as a highly heterogeneous and dynamic ecosystem rather than a static entity. Although conventional preclinical models often classify TAMs using a dichotomous M1/M2 paradigm, *in vivo* macrophage populations encompass highly diverse subsets, including Mhem and Mox, which distinctly express heme oxygenase-1 (HO-1) (Naito et al., 2014). Given the immunomodulatory effects of HO-1 and its metabolites, high-resolution techniques are required to elucidate whether APS and AS-IV selectively deplete these specific pro-tumorigenic subsets or facilitate their transition toward antigen-presenting states at a single-cell resolution. In a similar context, while experimental data indicate that APS can expand CD122^+^CXCR3^+^ memory T cells, advanced lineage tracing would help clarify whether these lymphocytes exist in a transient pre-exhaustion phase or have sustainably reverted to a durable effector phenotype. Beyond phenotypic diversity, spatial transcriptomics could provide critical insights into the topological distribution of these immune responses. While current evidence shows that these agents alter overall immune cell quantities, spatial profiling is necessary to verify whether they facilitate CD8^+^ T cell infiltration into hypoxic cores or if localized immunosuppressive niches—such as Treg and MDSC clusters—remain structurally intact.

Temporally, the immunomodulatory effects of APS and AS-IV likely evolve over the course of tumor progression, suggesting potential disparities between early and advanced stages of the disease. Although these compounds demonstrate efficacy across multiple preclinical models, it remains unclear if their primary cellular targets shift dynamically—for instance, predominantly modulating innate immune populations (e.g., macrophages and DCs) during early tumorigenesis, while later targeting MDSCs in advanced stages. Establishing longitudinal sampling paradigms across distinct stages of tumor growth is therefore a critical step toward clinical translation. Dynamic tracking of the TIME would allow for a more precise quantification of signaling kinetics, such as IFN-γ production, while also facilitating the identification of potentially evasive subpopulations—such as therapy-resistant Tregs or exhausted macrophages—that may emerge under therapeutic pressure. Ultimately, clarifying these spatiotemporal dynamics will offer an objective, evidence-based foundation for optimizing therapeutic windows and rationally designing combination strategies.

### 4.3 Overcoming translational barriers: advanced delivery systems, combinatorial strategies, and structural modifications

Translating the immunometabolic potential of APS and AS-IV into clinical interventions requires overcoming the complex physiological and microenvironmental barriers of the TME. While these compounds exhibit potent immunomodulatory activity *in vitro* and in animal models, their direct systemic deployment is frequently hindered by pharmacokinetic constraints, such as the low bioavailability of AS-IV and the vulnerability of APS to physiological degradation. To address these delivery challenges, spatiotemporally controlled nanoplatforms have been engineered, which demonstrate enhanced targeted tumor accumulation and robust anti-tumor immune responses in preclinical settings (Xu et al., 2022; Cao et al., 2024; Wang et al., 2024). For instance, in murine models, AS-IV liposomal nano-formulations utilize the mildly acidic TME to trigger the cGAS-STING pathway, thereby promoting CTL infiltration (Wang et al., 2024). Although these bio-responsive carriers demonstrate clear therapeutic efficacy, it remains mechanistically ambiguous whether the observed immune activation is driven entirely by the botanical payload, or if it partially represents a synergistic consequence of the carrier’s own physicochemical properties interacting with the TME.

Beyond delivery optimization, the co-administration of APS or AS-IV with immune checkpoint inhibitors (ICIs) represents a promising combinatorial approach. Experimental evidence indicates that APS can augment the efficacy of anti-PD-L1 therapy (Hwang et al., 2021), while AS-IV combined with anti-PD-1 blockade effectively suppresses tumor progression in murine models (Liu et al., 2020; Wu T. et al., 2024). By concurrently modulating metabolic pathways via natural products and alleviating immune suppression via checkpoint inhibitors, this regimen exhibits the potential to enhance immune infiltration in otherwise non-responsive tumors. Optimizing this synergistic efficacy, however, requires further clarity to determine whether APS and AS-IV specifically sensitize tumors to immune checkpoints or broadly alleviate microenvironmental stress. Consequently, the integration of artificial intelligence (AI) and network pharmacology may prove essential for rationally predicting optimal combinatorial regimens.

At the molecular level, modifying the structural composition of these agents through trace element incorporation provides an alternative translational pathway. *In vivo* studies indicate that Se-PAM exhibits enhanced efficacy in suppressing M2 macrophage polarization compared to its unmodified counterpart (Li et al., 2014). This observation suggests that incorporating trace elements (e.g., selenium and zinc) may favorably alter the spatial conformation or charge distribution of these molecules, thereby regulating their binding affinity to specific immune cell surface receptors. Evaluating the clinical viability of these structural modifications requires careful consideration. Because the precise structure-activity relationships of these macromolecular derivatives are not yet fully mapped, future endeavors should systematically explore how diverse elemental modifications influence both targeted immunomodulatory activities and long-term systemic clearance. Integrating these efforts with structural biology approaches may facilitate the transition of these agents from broad-spectrum empirical use toward rationally designed, mechanism-driven precision immunotherapy.

### 4.4 Translational considerations: biomarker stratification, advanced preclinical models, and pharmacological standardization

The potential clinical application of APS and AS-IV requires addressing the pronounced heterogeneity of the TIME. Given that immunological responses to these agents likely vary among diverse patient subpopulations, broad, unstratified administration may yield inconsistent clinical outcomes. Identifying specific baseline immunological parameters—such as the macrophage M1/M2 ratio or CD8^+^ T cell exhaustion markers (e.g., PD-1^+^ TIM-3^+^ expression)—could serve as a rational strategy to define potential responder populations. Prior to the initiation of biomarker-driven clinical trials, however, the efficacy of these targeted interventions requires rigorous validation in models that more accurately reflect the human immune landscape. While traditional 2D cell cultures provide foundational mechanistic insights, they often fail to capture the complex cellular cross-talk and metabolic architecture of the TME. Consequently, incorporating high-fidelity systems, such as PDOs and patient-derived xenografts (PDXs), represents a necessary methodological progression. By better preserving the original tumor’s immune composition and stromal scaffolding, these integrated models offer a more robust platform for evaluating potential synergistic interactions between APS/AS-IV and immune checkpoint blockades in a preclinical setting.

Beyond model selection, the pharmacological standardization of these botanical agents remains a critical hurdle for clinical translation. Variations in geographical origins, extraction protocols, and processing methodologies frequently induce fluctuations in the concentrations of active constituents. Although empirical applications of APS and AS-IV have demonstrated historical efficacy, this inherent chemical variability poses a significant challenge for maintaining therapeutic consistency. Addressing this translational gap will require the implementation of rigorous quality control systems, such as chromatographic fingerprinting, to accurately delineate characteristic peak clusters and improve batch-to-batch biological reproducibility. The development of such standardized formulations is a prerequisite for executing robust, multicenter, randomized, double-blind, placebo-controlled trials. Generating this level of high-quality evidence is ultimately necessary to evaluate whether these agents can successfully transition from traditional empirical use to rationally designed, precision-guided adjuvant therapies in oncology.

### 4.5 Limitations of current research

Although emerging preclinical evidence supports the immunomodulatory potential of APS and AS-IV, several knowledge gaps continue to impede their clinical translation. Current cellular investigations primarily emphasize classical immune subsets, leaving the pharmacological impact on type-2 skewed populations—such as N2 neutrophils, ILC2s, and NKT2 cells—largely uncharacterized. Specifically, experimental data demonstrating whether these compounds inhibit MMP secretion by N2 neutrophils or restrict ILC2 recruitment are currently lacking. Given that these subpopulations actively contribute to tumor-associated immunosuppression, incorporating them into future mechanistic models will be necessary to develop a comprehensive understanding of how *Astragalus* constituents remodel the TIME.

From a metabolic perspective, the existing literature is somewhat fragmented. Preliminary *in vitro* and *in vivo* studies highlight the modulatory effects of APS and AS-IV on key metabolic enzymes, including IDO, iNOS, and Arg-1 (Zhang et al., 2014; Wei et al., 2019; Ding et al., 2021; Wang et al., 2025). However, these observations are largely isolated to independent tryptophan and arginine pathways, lacking a unified framework that addresses potential metabolic crosstalk. Moreover, the exact mechanistic depth remains limited; it is not yet established whether these enzymes are direct binding targets of the compounds or if their altered expression reflects secondary adaptations to upstream signaling cascades. Validating these interactions through chemical biology techniques, such as Drug Affinity Responsive Target Stability (DARTS) or Surface Plasmon Resonance (SPR), will be critical to confirm the primary molecular engagement of APS and AS-IV.

Evaluating the spatiotemporal heterogeneity of the TME presents another major challenge. Many current methodologies rely on static endpoint assays, which may obscure how drug responses fluctuate across different stages of tumor progression or within distinct spatial niches, such as the invasive margin compared to the hypoxic core. Addressing these dynamic, time-dependent effects will be essential for optimizing therapeutic dosing schedules. Finally, the translation of APS and AS-IV into clinical practice is constrained by a lack of high-quality randomized controlled trials (RCTs) and established biomarker panels. Developing robust criteria for patient stratification, potentially based on baseline immune-metabolic profiling, represents a vital step for transitioning these botanical interventions from empirical use toward precision oncology.

### 4.6 Conclusions

In summary, APS and AS-IV exhibit significant potential as multifaceted immunometabolic modulators capable of shifting the TIME from an immunosuppressive state toward an immunostimulatory microenvironment. By regulating cellular immunity and disrupting tumor-driven bioenergetic competition, these compounds provide a rational framework to counteract immune evasion. Moving forward, integrating advanced nanodelivery platforms and combinatorial therapies with ICIs, alongside overcoming current pharmacological and methodological limitations, will be essential to successfully transition APS and AS-IV from empirical botanical extracts into rationally designed precision immunotherapies.
